# Phloroglucinol Derivatives from *Dryopteris crassirhizoma* as Potent Xanthine Oxidase Inhibitors

**DOI:** 10.3390/molecules26010122

**Published:** 2020-12-29

**Authors:** Heung Joo Yuk, Ji-Yul Kim, Yoon-Young Sung, Dong-Seon Kim

**Affiliations:** Herbal Medicine Research Division, Korea Institute of Oriental Medicine (KIOM), Daejeon 34054, Korea;yukhj@kiom.re.kr (H.J.Y.); jiyul2224@kiom.re.kr (J.-Y.K.); yysung@kiom.re.kr (Y.-Y.S.)

**Keywords:** *Dryopteris crassirhizoma*, gout, flavaspidic acid, phloroglucinols, traditional medicine, xanthine oxidase

## Abstract

*Dryopteris crassirhizoma* rhizomes are used as a traditional medicine in Asia. The EtOAc extract of these roots has shown potent xanthine oxidase (XO) inhibitory activity. However, the main phloroglucinols in *D. crassirhizoma* rhizomes have not been analyzed. Thus, we investigated the major constituents responsible for this effect. Bioassay-guided purification isolated four compounds: flavaspidic acid AP (**1**), flavaspidic acid AB (**2**), flavaspidic acid PB (**3**), and flavaspidic acid BB (**4**). Among these, **1** showed the most potent inhibitory activity with a half-maximal inhibitory concentration (IC_50_) value of 6.3 µM, similar to that of allopurinol (IC_50_ = 5.7 µM) and better than that of oxypurinol (IC_50_ = 43.1 µM), which are XO inhibitors. A comparative activity screen indicated that the acetyl group at C3 and C3′ is crucial for XO inhibition. For example, **1** showed nearly 4-fold higher efficacy than **4** (IC_50_ = 20.9 µM). Representative inhibitors (**1**–**4**) in the rhizomes of *D. crassirhizoma* showed reversible and noncompetitive inhibition toward XO. Furthermore, the potent inhibitors were shown to be present in high quantities in the rhizomes by a UPLC-QTOF-MS analysis. Therefore, the rhizomes of *D. crassirhizoma* could be used to develop nutraceuticals and medicines for the treatment of gout.

## 1. Introduction

Hyperuricemia is caused by the overproduction and/or underexcretion of uric acid [1,2]. The increased production of uric acid caused by the consumption of high-purine foods, uric acid metabolism disorders, and excessive purine breakdown also results in high uric acid levels in the blood [3]. Xanthine oxidase (XO), an essential enzyme in the body, is present in significant concentrations in the gastrointestinal tract and converts hypoxanthine and xanthine to uric acid in the purine catabolic pathway [4]. As a result, the increased uric acid levels cause the precipitation of urate crystals in the joints and kidneys, causing gout and gouty arthritis [5]. The use of allopurinol, a representative XO inhibitor that blocks uric acid synthesis in the body, is one of the therapeutic approaches for the clinical treatment of hyperuricemia and chronic gout [6]. However, it can cause a number of adverse side effects, such as allergic and hypersensitivity reactions, skin rash, gastrointestinal distress, renal failure, and renal toxicity [7]. Therefore, the development of potential XO-inhibitory agents from natural sources with greater efficacy, fewer side effects, and a better safety profile is highly desirable.

*Dryopteris crassirhizoma* Nakai *(Aspidiaceae*) is a perennial herbaceous plant that is distributed worldwide; its rhizomes have been used clinically in Korea, Japan, and China as a traditional medicine [8]. A previous study revealed that a crude 95% aqueous ethanol extract exerts antileukemic activity via cytotoxicity against the human leukemic Reh cell line [9]. There is also pharmacological evidence that this species possesses antiviral, anticancer, anti-obesity, and anti-melanogenic properties [10,11,12]. Chemical constituents in the rhizome of *D. crassirhizoma* have been reported to contain various compounds, including phloroglucinols, flavonoids, and triterpenes [13,14]. In particular, phloroglucinols, which are the main constituent, have various pharmacological properties, such as anti-inflammatory, antioxidant, and antibacterial activities [15,16]. Some studies have reported that compounds isolated from *D. crassirhizoma* rhizomes contribute to the biological activities; however, no detailed investigation of potential XO inhibitors has been carried out. Specifically, it is still unclear which compounds are the active ingredients in the extract, and the contributions of the main identified compounds to the XO-inhibitory activity of the extract are unknown. Additionally, no study has performed metabolite profiling and quantitative analyses using chromatographic techniques of the main phloroglucinols in *D. crassirhizoma* rhizomes.

In this study, we isolated and investigated the kinetics of the major constituents responsible for the XO-inhibitory effect of *D. crassirhizoma* rhizomes to support the use of the rhizome extract for the prevention and treatment of hyperuricemia. Additionally, these results may be used to suggest the use of the identified compounds as chemical markers for the quality assessment of *D. crassirhizoma* rhizomes.

## 2. Results and Discussion

### 2.1. Bioassay-Guided Isolation and Structural Identification of XO Inhibitors

**Figure 1:**
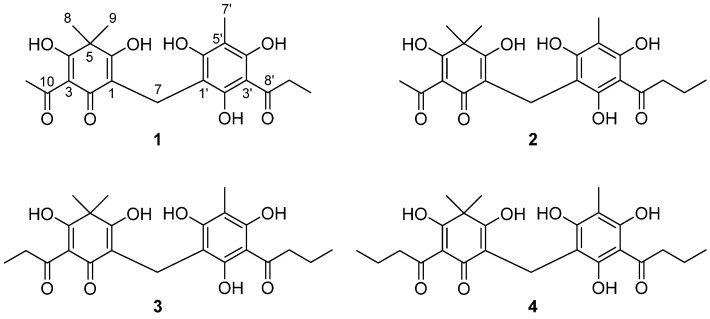
Chemical structures of isolated compounds **1**–**4** from rhizomes of *Dryopteris crassirhizoma*.

The extracts from four different polar solvents (EtOAc, MeOH, 50% MeOH, and H_2_O) were tested for their enzyme-inhibitory activities against XO. The enzyme assay followed the oxidation of xanthine to uric acid at 295 nm spectrophotometrically [17]. All extracts investigated, apart from the H_2_O extract, showed more than 50% inhibitory activity against XO at 150 μg/mL. In particular, EtOAc (half-maximal inhibitory concentration (IC_50_) = 48.7 μg/mL) was the solvent that yielded the maximum extraction of XO-inhibitory substances. The high potency of the EtOAc extract encouraged us to identify compounds responsible for its XO-inhibitory effects. The activity-guided fractionation of the EtOAc extract yielded four compounds, which were purified using octadecyl-functionalized silica gel (ODS) column chromatography (CC) and recycling preparative high-performance liquid chromatography (HPLC). The structures of isolated compounds (**1**–**4**; Figure 1) were elucidated using established spectroscopic analysis (1D/2D-nuclear magnetic resonance (NMR) and electrospray ionization high-resolution mass spectrometry (ESI-HR-MS), (Supplementary Materials Figures S1–S16) and through comparison with previously reported data [11,12,18]. Isolated compounds were identified as flavaspidic acid AP (**1**), flavaspidic acid AB (**2**), flavaspidic acid PB (**3**), and flavaspidic acid BB (**4**). Interestingly, the trace component flavaspidic acid BB (**4**) was first isolated by Lounasmaa in 1978 [18] but only C-NMR information was reported. In addition to ^13^C-NMR data, various spectroscopic data were used to identify the structures. Compound **4** was obtained as a yellow amorphous powder with the molecular formula C_24_H_30_O_8_ and 10 degrees of unsaturation established by ESI-HR-MS ([M − H]^−^ at *m/z* 445.1865, calcd. for C_24_H_29_O_8_ 445.1862, 0.7 ppm error). The ^13^C-NMR data enabled carbons corresponding to five C–C double bonds and three carbonyl groups to be identified, accounting for 8 of the 10 degrees of unsaturation. The extra two degrees of unsaturation were ascribed to two aromatic rings. The ^1^H-NMR spectrum showed the presence of five methylene groups (δ_H_ 3.45, 2H, s, H-7; δ_H_ 3.10, 2H, t, *J* = 7.3 Hz, H-9′; δ_H_ 2.82, 2H, t, *J* = 7.3 Hz, H-11; δ_H_ 1.69, 2H, m, H-10′; and δ_H_ 1.63, 2H, m, H-12) and five methyl groups (δ_H_ 1.95, 3H, s, H-7′; δ_H_ 1.24, 6H, s, H-8 and H-9; and δ_H_ 0.98, 6H, t, *J* = 7.3 Hz, H-13 and H-11′). They correlated to the carbons resonating at δ_C_ 47.3 (C-9′), δ_C_ 41.2 (C-11), δ_C_ 25.5 (C-8 and C-9), δ_C_ 20.8 (C-12), δ_C_ 19.8 (C-10′), δ_C_ 18.8 (C-7), δ_C_ 14.6 (C-11′ and C-13), and δ_C_ 8.0 (C-7′), respectively, in the heteronuclear multiple quantum coherence spectrum. The ^13^C-NMR spectrum showed 14 quaternary carbons, three carbonyl groups [δ_C_ 208.1 (C-8′), 197.1 (C-10), and 184.7 (C-2)], five oxygenated carbons [δ_C_ 202.5 (C-4), δ_C_ 196.9 (C-6), δ_C_ 162.9 (C-6′), δ_C_ 162.7 (C-4′), and 159.1 (C-2′)], and six quaternary carbons [δ_C_ 108.8 (C-1), δ_C_ 106.6 (C-1′), δ_C_ 106.2 (C-3), δ_C_ 105.3 (C-3′), δ_C_ 104.4 (C-5′), and δ_C_ 53.7 (C-5)]. These spectroscopic data are similar to those of flavaspidic acid PB (**3**), and the only difference is that flavaspidic acid BB (**4**) has a butyryl group instead of the propionyl group on the C-3 of compound **3**. The butyryl group on the C-3 of **4** was elucidated by the heteronuclear multiple bond correlations from H-13 to C-11 and C-12, H-12 to C-11 and C-13, and H-11 to C-10 and C-3. Thus, the structure of flavaspidic acid BB (**4**) was elucidated as 2-butyryl-6-(3-butyryl-2,4,6-trihydroxy-5-methylbenzyl)-3,5-dihydroxy-4,4-dimethylcyclohexa-2,5-dienone.

### 2.2. Contribution of the Identified Compounds to XO Inhibitory Activity

**Figure 2:**
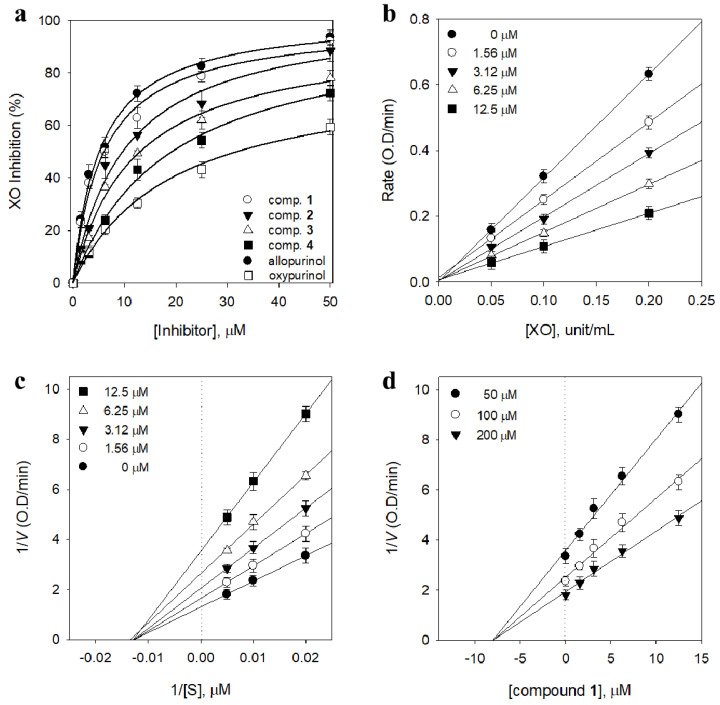
(**a**) Inhibitory effects of compounds **1**−**4** on the activity of xanthine oxidase (XO) based on the oxidation of xanthine to uric acid. (**b**) Catalytic activity of XO as a function of enzyme concentration at different concentrations of compound **1**. (**c**) Lineweaver–Burk plots were constructed for the inhibition of XO by compound **1**. The plot is expressed as 1/velocity versus 1/xanthine (S) with or without an inhibitor in the reaction solutions. (**d**) Dixon plots of XO inhibition by compound **1**. The graphical symbols are substrate concentrations (50 μM, ●; 100 μM, ○; 200 μM, ▼).

All isolated phloroglucinols (**1**–**4**) inhibited XO in a concentration-dependent manner with IC_50_ values ranging between 6.3 and 20.9 μM (Table 1, Figure 2A). Phloroglucinols have been reported to have several biological activities, including antioxidant, antiviral, and antibacterial effects [10,15,16]. However, to the best of our knowledge, the XO-inhibitory activity of the flavaspidic acid series of phloroglucinol derivatives is reported for the first time in this study. Among the four compounds, flavaspidic acid AP (**1**) exhibited the most potent inhibitory activity (79.6%) at a concentration of 25 μM, followed by compounds **2** (68.1%), **3** (62.1%), and **4** (54.4%). The potency of these inhibitors was affected by subtle changes in structure. It seemed that better inhibition was achieved with a polar substituent, in the following order: acetyl group (**2**, IC_50_ = 10.2 μM), propionyl group (**3**, IC_50_ = 13.2 μM), and butyryl group (**4**, IC_50_ = 20.9 μM) at the C-3 position. The potency of compound **1** (IC_50_ = 6.3 μM) can be favorably compared with that of selective XO inhibitors currently used as therapeutics, such as allopurinol (IC_50_ = 5.7 μM) [19]. To confirm the reversible inhibition caused by compound **1**, a plot was created in which the various inhibitor concentrations (0, 1.56, 3.12, 6.25, and 12.5 μM) versus their activity at different enzyme concentrations (0, 0.05, 0.1, and 0.2 units/mL) were analyzed. Increasing the inhibitor concentration resulted in a lowering of the line gradient, indicating that compound **1** is a reversible inhibitor (Figure 2B). We subsequently analyzed the type of inhibition using both Lineweaver–Burk (Figure 2C) and Dixon (Figure 2D) plots, which revealed that 1/y-intercept (*V*_max_) decreased, whereas –1/x-intercept (*K*_m_) remained constant, as the compound concentration increased. These results indicate that inhibitor **1** is a noncompetitive inhibitor, with an inhibition constant of 7.8 μM. Compounds **2**–**4** also displayed noncompetitive inhibition because increasing substrate concentrations resulted in a family of lines with different slopes but a common x-axis intercept. This noncompetitive behavior was also confirmed because *K*_m_ remained constant with an increasing concentration of inhibitors **2**−**4**, whereas *V*_max_ decreased (Supplementary Materials, Figures S17 and S18).

### 2.3. Ultra-Performance Liquid Chromatography (UPLC)-QTOF-MS Profiles

**Figure 3:**
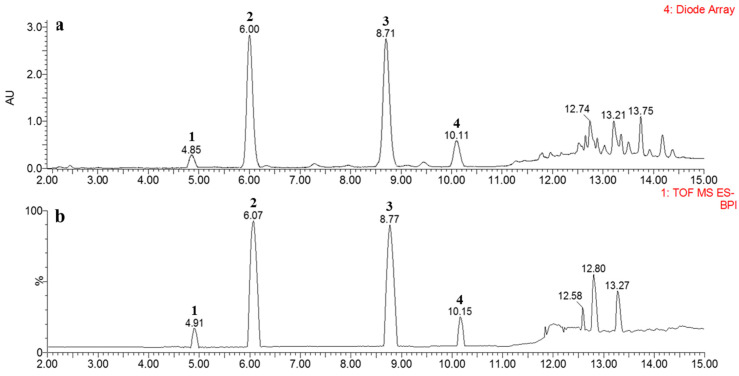
Representative chromatograms of EtOAc rhizome extract of *Dryopteris crassirhizoma*. (**a**) Diode array detector (DAD) chromatogram and (**b**) total ion current-base peak intensity (TIC-BPI) chromatogram.

**Figure 4:**
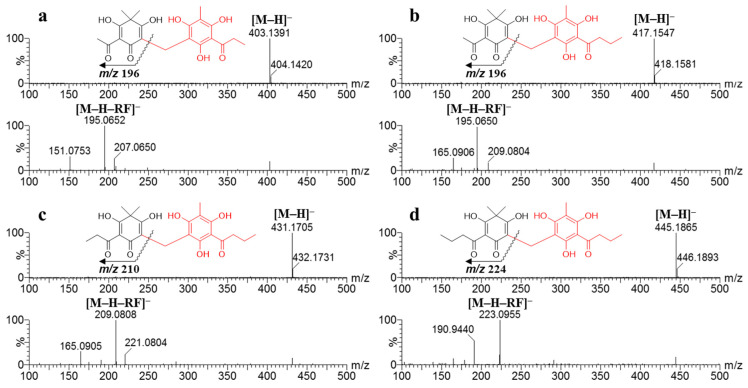
Negative ion mass spectrum acquired by UPLC-ESI/MS and MS/MS analysis of phloroglucinols (**a**–**d**, **1**–**4**, respectively) from rhizomes of *Dryopteris crassirhizoma*. Abbreviations: RF, residue of a fragment.

To establish the importance of the isolated compounds, a chemical profile of the constituents of the extracts from *D. crassirhizoma* using four different solvents was analyzed using UPLC-QTOF-MS. The EtOAc extract (11.3% of extraction yield), which showed potent inhibitory activity against XO, had a yield approximately half that of the MeOH extract (22.9% of extraction yield) but had the most abundant peaks of **1**–**4** (Table 1). The standard curves were linear and reproducible, as evidenced by the correlation coefficients (*r*^2^ = 0.998–0.999) for all isolated compounds. The levels of compounds **1**–**4** in the EtOAc extract were determined as 0.33, 4.41, 4.49, and 0.72 mg/g of dried sample, respectively (Table 2). In a preliminary study, extracts produced using MeOH, 50% MeOH, or H_2_O showed weak inhibitory effects on XO, and the levels of compounds **1**–**4** were also significantly lower than those in the EtOAc extract. The complete chromatographic separation of metabolites was achieved within 15 min (Figure 3). For each peak in the UPLC trace, the identity was verified by comparison with the retention time (*t*_R_) and the UV spectrum (*λ*_max_) of the isolated pure compound. All peaks also showed molecular ions with masses consistent with our obtained compounds (**1**[M − H]^−^ at *m/z* 403.1391; **2**, *m/z* 417.1547; **3**, *m/z* 431.1705; and **4**, *m/z* 445.1865). Four phloroglucinol derivatives were identified by QTOF-MS based on accurate mass, multiple-stage mass data, and characteristic fragmentation patterns (Figure 4). The main fragmentation peak represented the cleavage of the CH_2_ bridge group. The spectral characteristics for the identification of the phloroglucinol derivatives, including retention times, UV *λ*_max_, accurate mass, and elemental composition, are summarized in Table 2.

## 3. Materials and Methods

### 3.1. Plant Materials and Sample Preparation

The rhizomes of *D. crassirhizoma* cultivated in Uiseong-gun (Gyeongsangbuk-do, Republic of Korea) were purchased from an oriental herbal market (Omniherb, Daegu metropolitan city, Republic of Korea) (http://www.omniherb.com/), which only handles herbs certified by the Korean Pharmacopoeia. The dried rhizomes were ground to a powder and stored at –75 °C until further analysis. Four different solvent extraction systems were used: EtOAc, MeOH, 50% MeOH in water (50% MeOH, MeOH/H_2_O (1/1, *v*/*v*)), and distilled H_2_O. All samples were sonicated twice for 30 min at 25 °C, were filtered through a 0.2 μm polytetrafluoroethylene filter, and used for enzymatic assays and liquid chromatography analyses. All extraction and chromatographic solvents were HPLC grade (J. T. Baker, Phillipsburg, NJ, USA). Trifluoroacetic acid (TFA) (Sigma-Aldrich, St. Louis, MO, USA) was used to control the pH of the chromatographic mobile phase.

### 3.2. Instruments

The ^1^H and ^13^C-NMR spectra were recorded on a JNM-ECZ600R FT-NMR spectrometer (JEOL, Tokyo, Japan), using CD_3_OD with tetramethylsilane as the internal standard (Andover, MA, USA). Melting points (mp) were measured on a Thomas Scientific Capillary Melting Point Apparatus (Swedesboro, NJ, USA) and were uncorrected. The IR spectra were recorded on a Perkin Elmer Frontier FT-IR spectrophotometer (PerkinElmer, Waltham, MA, USA). ESI-HR mass spectra were obtained on a quadrupole time of flight mass spectrometer (Xevo G2-S QTOF, Waters Corp., Milford, MA, USA). The UPLC system, equipped with a binary solvent delivery system, an auto-sampler, and a UV detector, was also obtained from Waters Corp. Separation was performed using a medium-pressure liquid chromatography (MPLC) system (LC-forte/R; YMC Co., Ltd., Kyoto, Japan) with reversed-phase (RP) cartridges. Purification was carried out on a recycling HPLC system (LC-200 NEXT; Japan Analytical Industry Co., Ltd., Tokyo, Japan), with which a GS-310 (500 mm × 20 mm, i.d. 20 µm) column purchased from Japan Analytical Industry (JAI) was employed. Enzymatic assays were carried out on a SpectraMax Multi-Mode Microplate Reader M2 (Molecular Devices, San Jose, CA, USA).

### 3.3. Sample Extraction, Fractionation, and Isolation

Dried rhizomes (1 kg) of *D. crassirhizoma* were powdered using a laboratory blade cutter and extracted with MeOH (18 L × 2) at room temperature. The combined extract was evaporated, resulting in 180 g of crude extract. In the preliminary experiment, the EtOAc extract had high inhibitory activity against XO, but the extraction yield was only half that of the MeOH extract; thus, the active substance was extracted with MeOH, suspended in H_2_O, and partitioned with EtOAc. MeOH is the best solvent for the maximum extraction of secondary metabolites from natural sources and foods [20]. The partitioned EtOAc extract (50 g) was subjected to CC on ODS (12 × 40 cm, 1000 g) and eluted with MeOH/H_2_O mixtures [30:70 (2 L), 50:50 (2 L), 70:30 (4 L), 90:10 (2 L), and 100:0 (2 L)] to yield five fractions (A–E). Fraction C (5.2 g) was fractionated on an RP column (220 g, C18 cartridge) using MPLC with a linear gradient of 60–80% MeOH/H_2_O and a flow rate of 20 mL/min to afford five fractions (C1–C5). Subfractions (C2–C3), enriched with compounds **1** and **2**, were combined (740 mg) and further purified by recycling preparative HPLC using 70% mixed solvents (MeOH/propanol (8/2, *v*/*v*)) in H_2_O as the mobile phase, which produced compounds **1** (24 mg) and **2** (143 mg). Fraction D (4.8 g) was fractionated via RP-MPLC using a C18 column cartridge with elution using a gradient of increasing MeOH (70–90%) in H_2_O to yield fractions D1–D7. Subfractions (D3–C6), enriched with compounds **3** and **4**, were combined (625 mg) and further purified by recycling preparative HPLC using 70% mixed solvents (MeOH/propanol (8/2, *v*/*v*)) in H_2_O as the mobile phase, which produced compounds **3** (127 mg) and **4** (34 mg). The physicochemical and spectrometric data of the four compounds (**1**–**4**) are as follows:

*Flavaspidic acid AP* (**1**). Yellow amorphous powder; mp 158–160 °C; IR (ATR) *v*_max_ 3388.64 (OH), 2933.41 (CH), 1613.32 (C=O), 1485.54 (C=C), 1161.63, 1120.49 (C–O) cm^–1^; negative ESI-HR-MS, *m/z*: 403.1391 [M − H]^−^ (calcd. for C_21_H_23_O_8_ 403.1393); ^1^H-NMR (600 MHz, CD_3_OD): δ 3.45 (2H, s, H-7), δ 3.15 (2H, t, *J* = 7.3 Hz, H-9′), δ 2.43 (3H, s, H-11), δ 1.95 (3H, s, H-7′), δ 1.25 (6H, s, H-8 and H-9), δ 1.14 (3H, t, *J* = 7.3 Hz, H-10′); ^13^C-NMR (125 MHz, CD_3_OD): δ 208.8 (C-8′), δ 202.7 (C-4), δ 197.3 (C-6), δ 194.3 (C-10), δ 184.2 (C-2), δ 162.9 (C-6′), δ 162.6 (C-4′), δ 159.1 (C-2ʹ), δ 108.7 (C-1), δ 106.4 (C-1′), δ 106.0 (C-3′), δ 105.6 (C-3), δ 104.4 (C-5′), δ 53.6 (C-5), δ 38.4 (C-9′), δ 26.8 (C-11), δ 25.5 (C-8 and C-9), δ 18.7 (C-7), δ 9.7 (C-10ʹ), δ 8.0. (C-7′).

*Flavaspidic acid AB* (**2**). Yellow amorphous powder; mp 158–160 °C; IR (ATR) *v*_max_ 3287.94 (OH), 2931.22 (CH), 1612.63 (C=O), 1482.38 (C=C), 1162.50, 1124.24 (C–O) cm^–1^; negative ESI-HR-MS, *m/z*: 417.1547 [M − H]^−^ (calcd. for C_22_H_25_O_8_ 417.1549); ^1^H-NMR (600 MHz, CD_3_OD): δ 3.45 (2H, s, H-7), δ 3.10 (2H, t, *J* = 7.3 Hz, H-9′), δ 2.43 (3H, s, H-11), δ 1.95 (3H, s, H-7′), δ 1.69 (2H, m, H-10′), δ 1.25 (6H, s, H-8 and H-9), δ 0.98 (3H, t, *J* = 7.3 Hz, H-11′); ^13^C-NMR (125 MHz, CD_3_OD): δ 208.2 (C-8′), δ 202.7 (C-4), δ 197.3 (C-6), δ 194.3 (C-10), δ 184.1 (C-2), δ 163.0 (C-6′), δ 162.7 (C-4′), δ 159.1 (C-2′), δ 108.7 (C-1), δ 106.4 (C-1′), δ 106.2 (C-3′), δ 105.6 (C-3), δ 104.4 (C-5′), δ 53.6 (C-5), δ 47.3 (C-9′), δ 26.8 (C-11), δ 25.5 (C-8 and C-9), δ 19.8 (C-10ʹ), δ 18.7 (C-7), δ 14.5 (C-11′), δ 8.0. (C-7′).

*Flavaspidic acid PB* (**3**). Yellow amorphous powder; mp 156–158 °C; IR (ATR) *v*_max_ 3254.12 (OH), 2934.34 (CH), 1610.52 (C=O), 1475.56 (C=C), 1158.84, 1123.26 (C–O) cm^–1^; negative ESI-HR-MS, *m/z*: 431.1705 [M − H]^−^ (calcd. for C_23_H_27_O_8_ 431.1706); ^1^H-NMR (600 MHz, CD_3_OD): δ 3.46 (2H, s, H-7), δ 3.11 (2H, t, *J* = 7.3 Hz, H-9′), δ 2.87 (2H, m, H-11), δ 1.95 (3H, s, H-7′), δ 1.69 (2H, m, H-10′), 1.25 δ (6H, s, H-8 and H-9), δ 1.14 (3H, t, *J* = 7.3 Hz, H-12), δ 0.99 (3H, t, *J* = 7.3 Hz, H-11′); ^13^C-NMR (125 MHz, CD_3_OD): δ 208.2 (C-8′), δ 202.4 (C-4), δ 198.5 (C-10), δ 197.1 (C-6), δ 184.6 (C-2), δ 163.0 (C-6′), δ 162.7 (C-4′), δ 159.1 (C-2′), δ 108.8 (C-1), δ 106.5 (C-1′), δ 106.2 (C-3), δ 104.8 (C-3′), δ 104.4 (C-5′), δ 53.7 (C-5), δ 47.3 (C-8′), δ 32.7 (C-11), δ 25.5 (C-8 and C-9), δ 19.9 (C-10′), δ 18.8 (C-7), δ 14.6 (C-11′), δ 10.5 (C-12), δ 8.0 (C-7′).

*Flavaspidic acid BB* (**4**). Yellow amorphous powder; mp 156–158 °C; IR (ATR) *v*_max_ 3238.92 (OH), 2932.21 (CH), 1612.79 (C=O), 1488.07 (C=C), 1159.55, 1124.29 (C–O) cm^–1^; negative ESI-HR-MS, *m/z*: 445.1865 [M − H]^−^ (calcd for C_24_H_29_O_8_ 445.1862); ^1^H-NMR (600 MHz, CD_3_OD): δ 3.45 (2H, s, H-7), δ 3.10 (2H, t, *J* = 7.3 Hz, H-9′), δ 2.82 (2H, t, *J* = 7.3 Hz, H-11), δ 1.95 (3H, s, H-7′), δ 1.69 (2H, m, H-10′), δ 1.63 (2H, m, H-12), δ 1.24 (6H, s, H-8 and H-9), δ 0.98 (6H, t, *J* = 7.3 Hz, H-13 and H-11′); ^13^C-NMR (125 MHz, CD_3_OD): δ 208.1 (C-8′), δ 202.5 (C-4), δ 197.1 (C-10), δ 196.9 (C-6), δ 184.7 (C-2), δ 162.9 (C-6′), δ 162.7 (C-4′), δ 159.1 (C-2′), δ 108.8 (C-1), δ 106.6 (C-1′), δ 106.2 (C-3), δ 105.3 (C-3′), δ 104.4 (C-5′), δ 53.7 (C-5), δ 47.3 (C-9′), δ 41.2 (C-11), δ 25.5 (C-8 and C-9), δ 20.8 (C-12), δ 19.8 (C-10′), δ 18.8 (C-7), δ 14.6 (C-11′ and C-13), δ 8.0 (C-7′).

### 3.4. UPLC-QTOF-MS Analysis

UPLC was performed on a Waters Acquity UPLC system coupled with a QTOF ESI/mass spectrometer. Aliquots (3.0 μL) of each sample were injected into a BEH C18 column (100 × 2.1 mm, i.d., 1.7 μm) at a flow rate of 0.3 mL/min and eluted using a chromatographic gradient of two mobile phases (A: water containing 0.1% TFA; B: MeOH/propanol (8/2, *v*/*v*) containing 0.1% TFA). A linear gradient was optimized as follows: 0 min, 70% B; 0–1 min, 70% B; 1–10.3 min, 70–100% B; 10.3–13.3 min, 100% B; 13.3–13.4 min, 100–70% B; 13.4–15 min, stabilized to 70% B. The QTOF spectrometer was operated in negative-ion mode in the following conditions: capillary voltage, 2.3 kV; cone voltage, 50 V; source temperature, 110 °C; and desolvation temperature, 350 °C. A sprayer with a reference solution of leucine-enkephalin ([M − H]^−^ *m/z* 554.2615) was used as the lock mass. The full scan data and MS/MS spectra were collected using MassLynx 4.1 software (Waters Corporation, Milford, MA, USA).

### 3.5. Assay of XO-Inhibitory Activity and Kinetics

XO-inhibitory activity was assayed spectrophotometrically on a SpectraMax M2 microplate reader, using a previously reported experimental procedure with a slight modification [21]. First, 135 µL of 100 mM sodium pyrophosphate buffer (HCl, pH 7.5), 20 µL of 0.1 unit XO enzyme (bovine milk) in buffer, and 5 µL of samples (test extracts or compounds) in dimethylsulfoxide were mixed at 37 °C. The reaction was started by adding 40 µL of substrate (0.5 mM xanthine) in buffer to the mixture. The reaction mixture (200 µL) was incubated at 37 °C in a 300 µL well plate and the UV absorbance at 295 nm was determined every 1 min up to 5 min. Allopurinol and oxypurinol served as positive controls.

### 3.6. Statistical Analysis

All measurements of in vitro inhibitory activity (XO inhibition and kinetic parameters) and compound contents were performed at least in triplicate and data are expressed as mean ± SD (standard deviation) using Sigma plot 10.0 (Systat Software Inc., San Jose, CA, USA).

## 4. Conclusions

The rhizome extract of *D. crassirhizoma* has a significant inhibitory effect on XO in vitro. In this study, for the first time, the potent inhibitors were identified as flavaspidic acid AP (**1**) and its derivatives (**2**−**4**), with inhibitory activity against XO at low micromolar concentrations (IC_50_ = 6.3−20.9 μM). Based on the detailed kinetic analysis of the most potent inhibitor using double-reciprocal plots, **1** was determined to be a noncompetitive inhibitor (*K*_i_ = 7.8 μM). The sum of the identified phloroglucinol derivatives was approximately 10 mg/g of dried sample, and we believe that the potent XO-inhibitory activity of the extract was related to its derivatives. Additionally, a simple, rapid, and sensitive UPLC method was developed for the analysis of phloroglucinol derivatives. Overall, these results suggest that the rhizomes of *D. crassirhizoma* could be used for the development of nutraceuticals and medicines for the treatment of gout.

## Figures and Tables

**Table 1:** Inhibitory effects of the rhizome extract of *Dryopteris crassirhizoma* using different solvents and isolated compounds **1**–**4** on xanthine oxidase activities.

Compound	Extraction Yield (%) ^1^	Xanthine Oxidase		
		IC_50_^2^	Inhibition % ^3^	Kinetic Mode (*K_i_* ^4^, µM)
EtOAc	11.3	48.7 ± 1.4 ppm	76.5 ± 1.5	NT^5^
MeOH	22.9	54.4 ± 1.1 ppm	72.4 ± 1.3	NT
50% MeOH	21.6	134.5 ± 1.7 ppm	46.8 ± 1.2	NT
H_2_O	14.5	>500 ppm	7.5 ± 1.0	NT
**1**	-	6.3 ± 0.4 µM	79.6 ± 1.6	Noncompetitive (7.8)
**2**	-	10.2 ± 0.5 µM	68.1 ± 1.3	Noncompetitive (11.7)
**3**	-	13.2 ± 0.6 µM	62.1 ± 0.9	Noncompetitive (15.3)
**4**	-	20.9 ± 0.9 µM	54.4 ± 1.5	Noncompetitive (21.1)
Allopurinol	-	5.7 ± 0.2 µM	82.5 ± 1.7	Competitive
Oxypurinol	-	43.1 ± 0.8 µM	42.2 ± 1.2	NT

All extracts and compounds were examined in a set of experiments repeated three times. ^1^ Extraction yields are given as mg/g dry weight; ^2^ IC_50_ values of compounds represent the concentration that caused 50% enzyme activity loss; ^3^ sample concentration was 125 ppm (µg/mL) for the extract and 25 µM for each compound; ^4^ values of inhibition constant; NT: not tested.

**Table 2:** Spectral characteristics and contents (mg/g) of the four investigated compounds in rhizomes of *Dryopteris crassirhizoma*.

Peak	*t*_R_	*λ*_max_	Dried Rhizomes (mg/g) ^1^				[M − H]^−^ (*m/z*)	Molecular Formula	Identification
(no.)	(min)	(nm)	EtOAc	MeOH	50%MeOH	H_2_O	(ESI-HR-MS)	(ppm Error)	
1	4.85	294	0.33	0.32	0.21	tr	403.1391	C_21_H_23_O_8_ (−0.5)	Flavaspidic acid AP
2	6.00	296	4.41	4.12	3.46	tr	417.1547	C_22_H_25_O_8_ (−0.5)	Flavaspidic acid AB
3	8.71	296	4.49	4.47	3.26	tr	431.1705	C_23_H_27_O_8_ (−0.2)	Flavaspidic acid PB
4	10.11	295	0.72	0.71	0.56	tr	445.1865	C_24_H_29_O_8_ (0.7)	Flavaspidic acid BB

^1^ All values are expressed as mean (*n* = 3), content expressed as milligrams of each compound equivalents per gram of dry weight.

## Data Availability

The data presented in this study are available in article and supplementary materials here.

## Supplementary Materials

The following are available online: Figures S1–S4: ^1^H-NMR spectrum of compounds **1**−**4**, Figures S5–S8: ^13^C-NMR spectrum of compounds **1**−**4, Figures S9–S12:** IR Spectrum of compounds **1-4**, Figures S13–S16: Identification of compounds **1**−**4** by UPLC-QTOF-MS, Figures S17 and S18: Kinetics analysis of compounds **1**–**4**.

## Sample Availability

Samples of the compounds are not available from the authors.

## References

[1] Gibson T. (2012). Hyperuricemia, gout and the kidney. Curr. Opin. Rheumatol..

[2] Bitik B., Öztürk M.A. (2014). An old disease with new insights: Update on diagnosis and treatment of gout. Eur. J. Rheumatol..

[3] Fan H., Zheng A., Xu P., Wang J., Xue T., Dai S., Pan S., Guo Y., Xie X., Li L. (2020). High-protein diet induces hyperuricemia in a new animal model for studying human gout. Int. J. Mol. Sci..

[4] Fukunari A., Okamoto K., Nishino T.B., Eger T., Pai E.F., Kamezawa M., Yamada I., Kato N. (2004). Y-700 [1-[3-Cyano-4-(2,2-dimethylpropoxy)phenyl]-1*H*-pyrazole-4-carboxylic acid]: A potent xanthine oxidoreductase inhibitor with hepatic excretion. J. Pharmacol. Exp. Ther..

[5] Martillo M.A., Nazzal L., Crittenden D.B. (2014). The crystallization of monosodium urate. Curr. Rheumatol. Rep..

[6] Huo L.-N., Wang W., Zhang C.-Y., Shi H.-B., Liu Y., Liu X.H., Guo B.-H., Zhao D.-M., Gao H. (2015). Bioassy-guided isolation and identification of xanthine oxidase inhibitory constituents from the leaves of *Perilla frutescens*. Molecules.

[7] Pacher P., Nivorozhkin A., Szabo C. (2006). Therapeutic effects of xanthine oxidase inhibitor: Renaissance half a century after the discovery of allopurinol. Pharmacol. Rev..

[8] Namba T. (1994). The Encyclopedia of Wakan-Yaku (Traditional Sino-Japanese Medicines) with Color Pictures.

[9] Ren Q., Quan X.-G., Wang Y.-L., Wang H.-Y. (2016). Isolation and identification of phloroglucinol derivatives from *Dryopteris crassirhizoma* by HPLC-LTQ-Orbitrap Mass Spectrometry. Chem. Nat. Compd..

[10] Wang J., Yan Y.-T., Fu S.-Z., Peng B., Bao L.-L., Zhang Y.-L., Hu J.-H., Zeng Z.-P., Geng D.-H., Gao Z.P. (2017). Anti-influenza virus (H5N1) activity screening on the phloroglucinols from rhizomes of *Dryopteris crassirhizoma*. Molecules.

[11] Na M.-K., Jang J.-P., Min B.-S., Lee S.-J., Lee M.S., Kim B.-Y., Oh W.-K., Ahn J.-S. (2016). Fatty acid synthase inhibitory activity of acylphloroglucinols isolated from *Dryopteris crassirhizoma*. Bioorg. Med. Chem. Lett..

[12] Pham V.-C., Kim O.-H., Lee J.-H., Min B.-S., Kim J.-A. (2017). Inhibitory effects of phloroglucinols from the roots of *Dryopteris crassirhizoma* on melanogenesis. Phytochem. Lett..

[13] Gao Z.-P., Ali Z., Zhao J.-P., Qiao L., Lei H.-M., Lu Y.R., Khan I.-A. (2008). Phytochemical investigation of the rhizomes of *Dryopteris crassirhizoma*. Phytochem. Lett..

[14] Yang Y.-Y., Lee G.-J., Yoon D.-H., Yu T., Oh J.-E., Jeong D.-O., Lee J.-S., Kim S.-H., Kim T., Cho J.Y. (2013). ERK_1_- and TBK_1_-targeted anti-inflammatory activity of an ethanol extract of *Dryopteris crassirhizoma*. Phytochem. Lett..

[15] Lee S.-M., Na M.-K., Na R.-B., Min B.-S., Lee H.-K. (2003). Antioxidant activity of two phloroglucinol derivatives from *Dryopteris crassirhizoma*. Biol. Pharm. Bull..

[16] Lee H., Kim J.-C., Lee S.-M. (2009). Antibacterial activity of two phloroglucinols, flavaspidic acids AB and PB, from *Dryopteris crassirhizoma*. Arch. Pharm. Res..

[17] Liu K., Wang W., Guo B.-H., Gao H., Liu Y., Liu X.-H., Yao H.-L., Cheng K. (2016). Chemical evidence for potent xanthine oxidase inhibitory activity of ethyl acetate extract of *Citrus aurantium* L. dried immature fruits. Molecules.

[18] Lounasmaa M. (1978). Dérivés Phloroglucinoliques des Fougères du Genre Dryopteris. Planta Med..

[19] Chu Y.-H., Chen C.-J., Wu S.-H., Hsieh J.-F. (2014). Inhibition of xanthine oxidase by *Rhodiola crenulata* extracts and their phytochemicals. J. Agric. Food Chem..

[20] Wang Y., Yuk H.-J., Kim J.-Y., Kim D.-W., Song Y.-H., Tan X.-F., Curtis-Long M.-J., Park K.-H. (2016). Novel chromenedione derivatives displaying inhibition of protein tyrosine phosphatase 1B (PTP1B) from *Flemingia philippinensis*. Bioorg. Med. Chem. Lett..

[21] Yuk H.-J., Lee Y.-S., Ryu H.-W., Kim S.-H., Kim D.-S. (2018). Effects of *Toona sinensis* leaf extract and its chemical constituents on xanthine oxidase activity and serum uric acid levels in potassium oxonate-induced hyperuricemic rats. Molecules.

