# Supplementary material for: Phloroglucinol Derivatives from Dryopteris crassirhizoma as Potent Xanthine Oxidase Inhibitors
Source: Molecules. 2020 Dec 29;26(1):122. doi: 10.3390/molecules26010122 (PMC7796287; doi:10.3390/molecules26010122)
Supplement: Supplementary file 1 [file molecules-26-00122-s001.pdf]

Supplementary Materials

Phloroglucinol derivatives from *Dryopteris crassirhizoma* as potent xanthine oxidase inhibitors

Heung Joo Yuk, Ji-Yul Kim, Yoon-Young Sung and Dong-Seon Kim*

Title page ……………………………………………………………………………… 1

Figure S1−4. ^{1}H-NMR spectrum of compounds (**1**−**4**) ……………………………………… 2−5

Figure S5−8. ^{13}C-NMR spectrum of compounds (**1**−**4**) ……………………………………… 6−9

Figure S9−12. IR spectrum of compounds (**1**−**4**) ……………………………………………… 10−13

Figure S13−16. Identification of compounds (**1**−**4**) by UPLC-Q-TOF MS ……………………… 14−17

Figure S17−18. Kinetics analysis of compounds (**1**−**4**) ………………………………………… 18

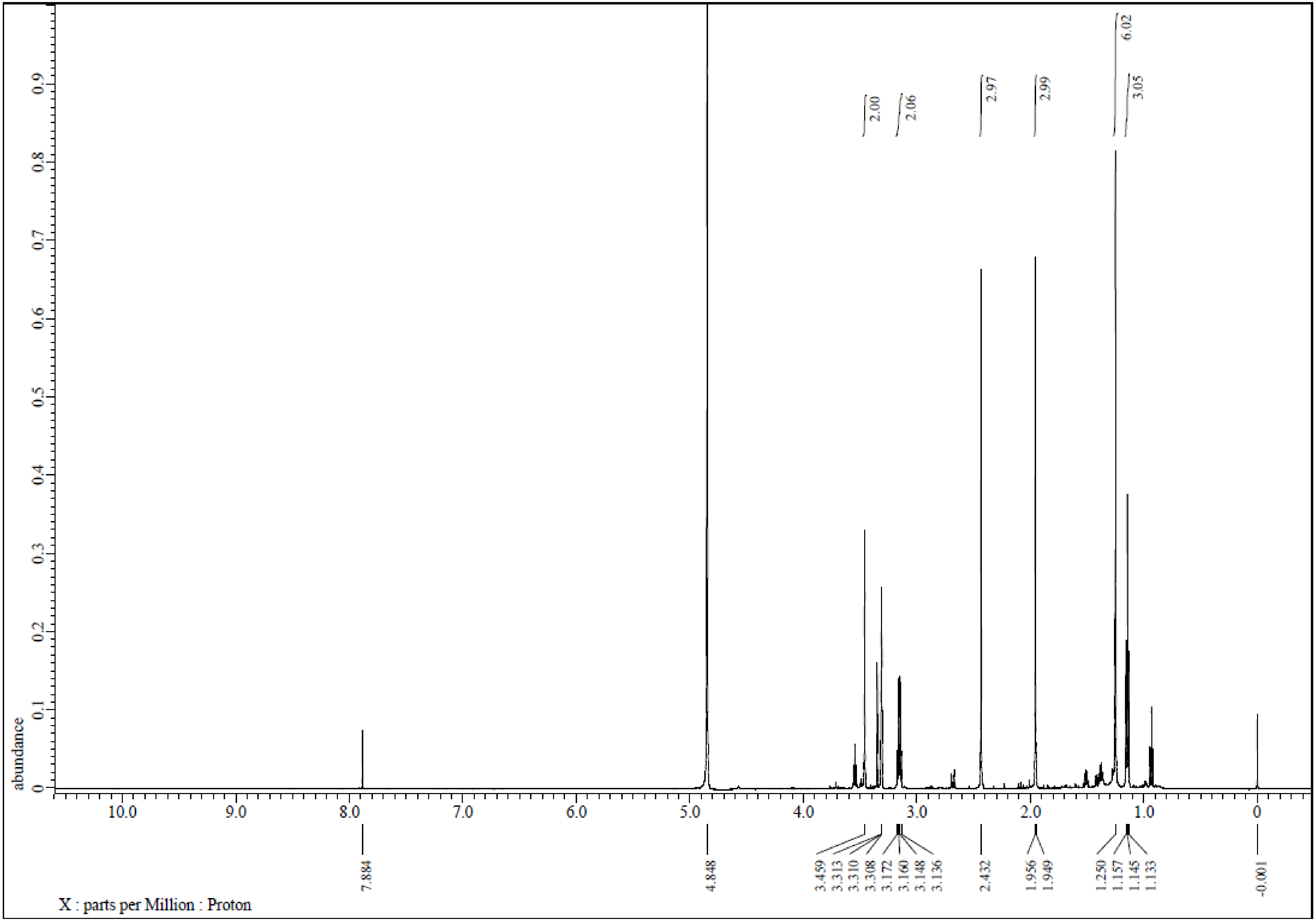

Figure S1. The ^{1}H NMR spectrum of compound **1** (600 MHz, CD_3OD).

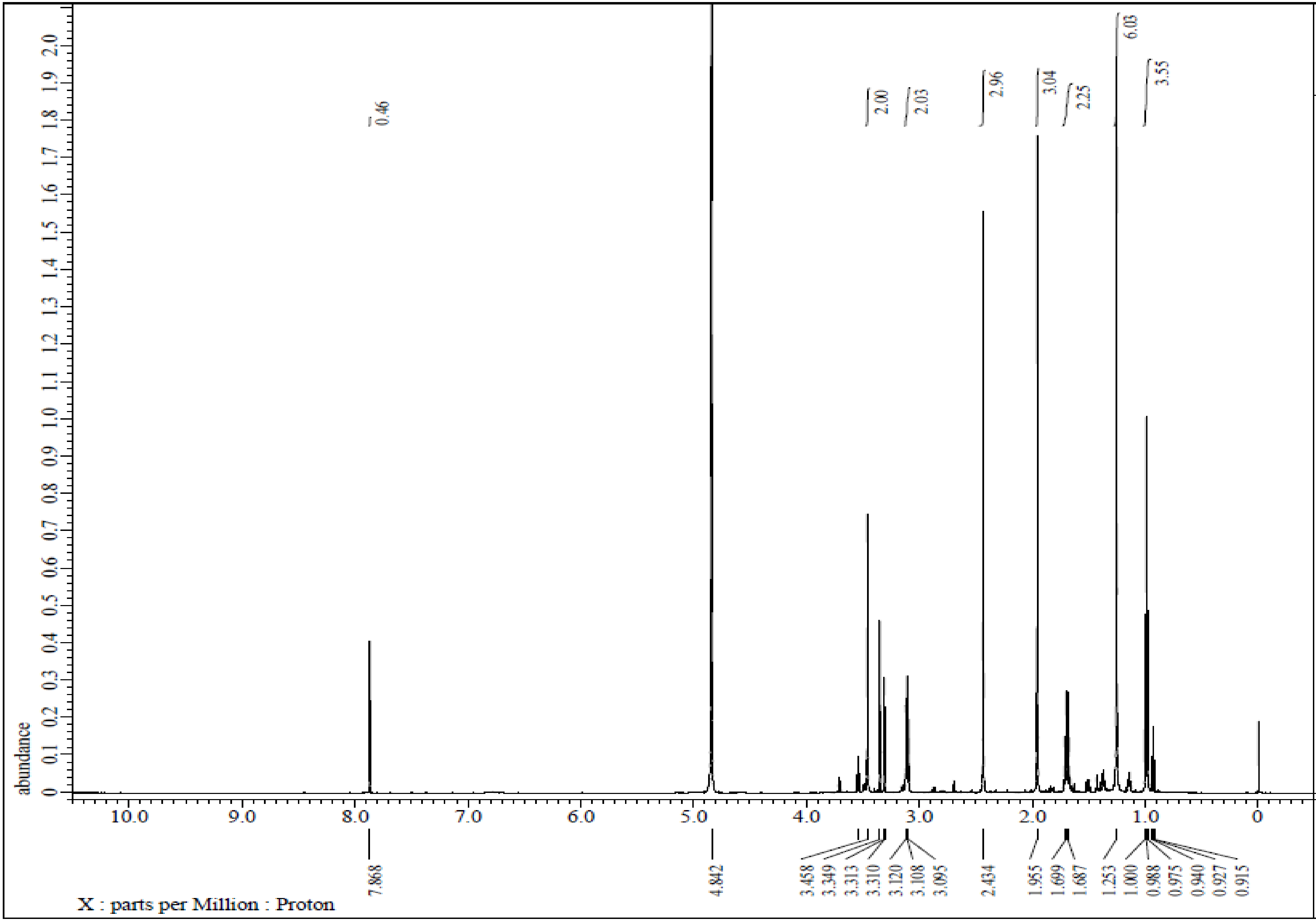

Figure S2. The ^{1}H NMR spectrum of compound **2** (600 MHz, CD_3OD).

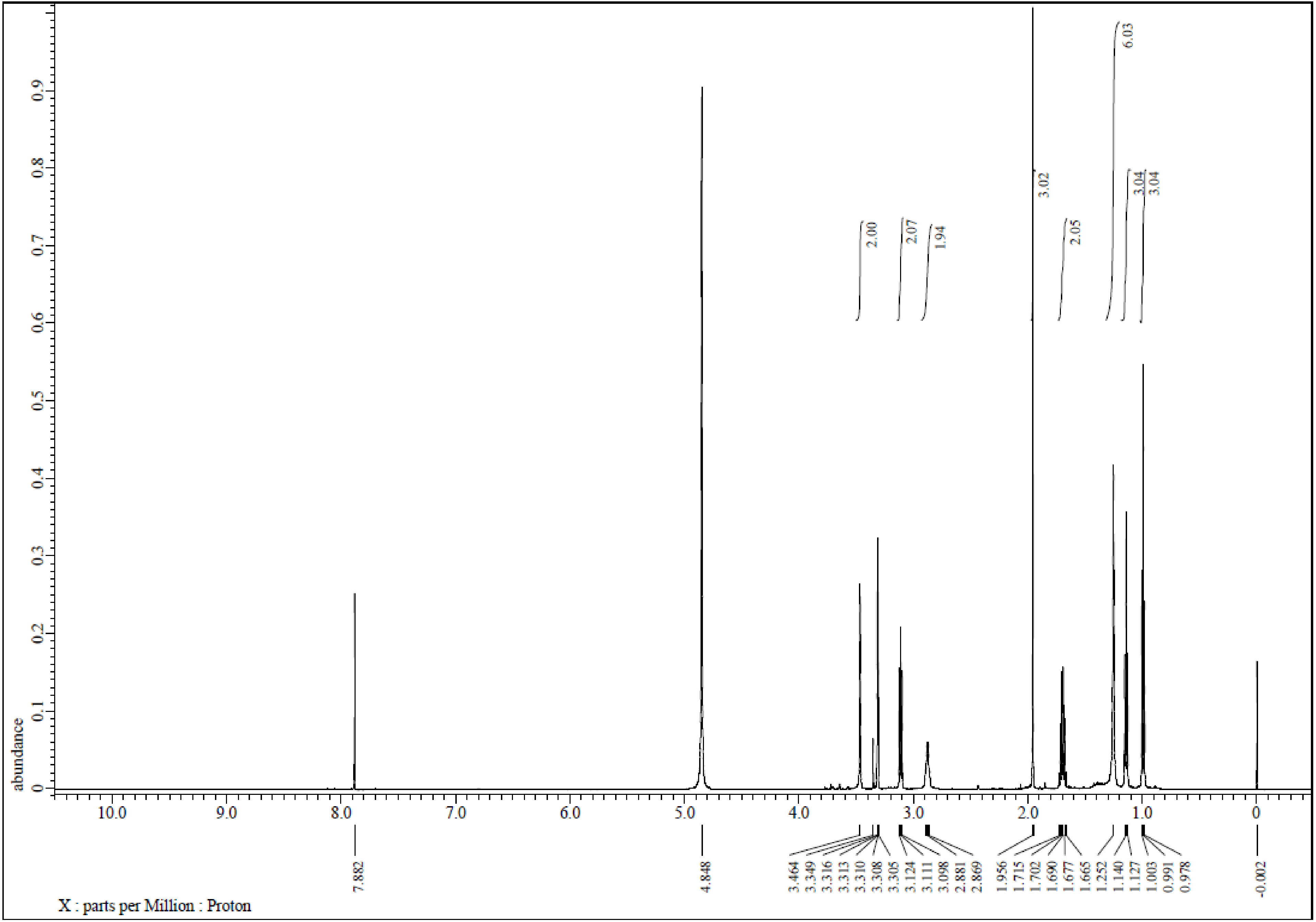

Figure S3. The ^{1}H NMR spectrum of compound **3** (600 MHz, CD_3OD).

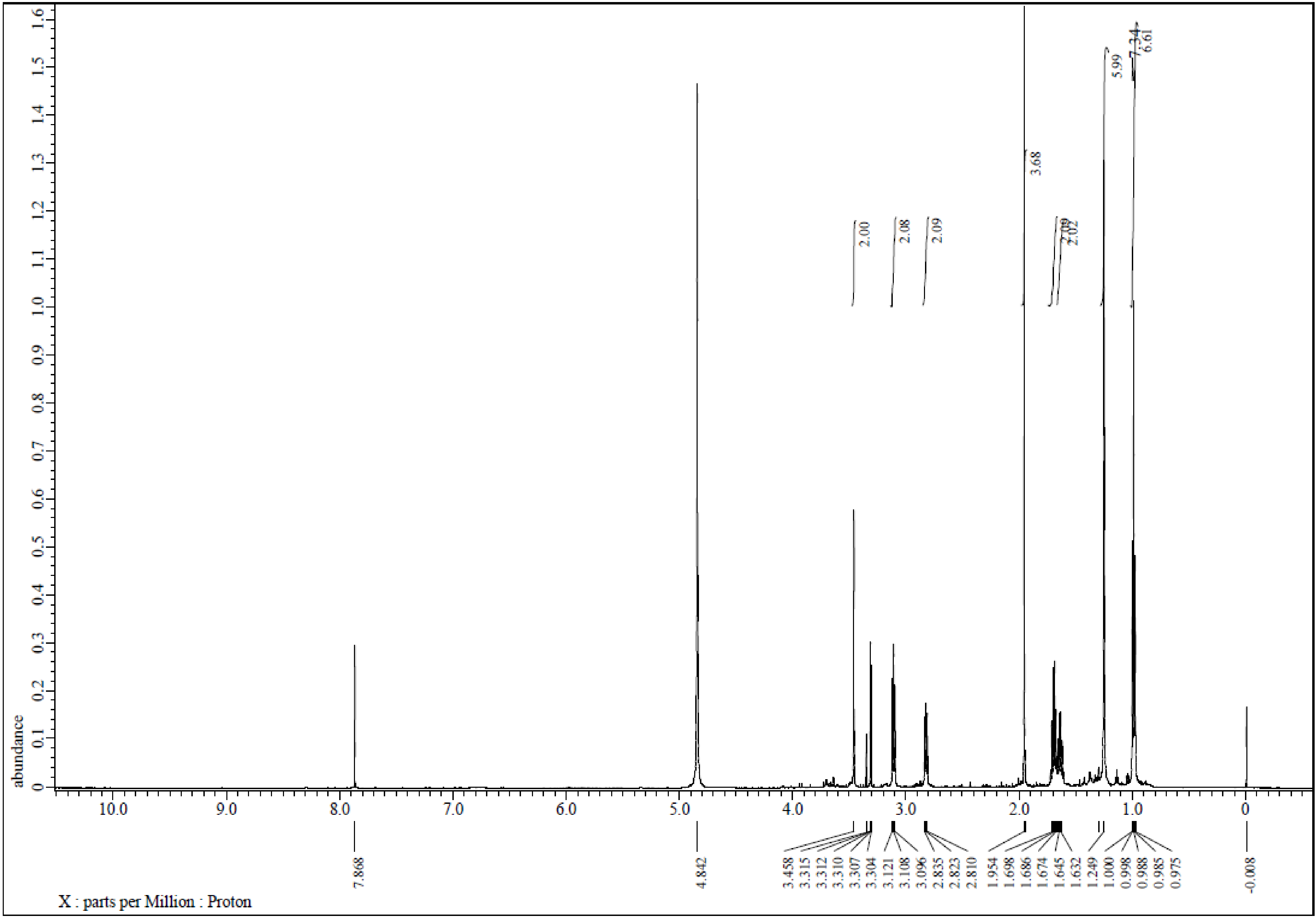

Figure S4. The ^{1}H NMR spectrum of compound **4** (600 MHz, CD_3OD).

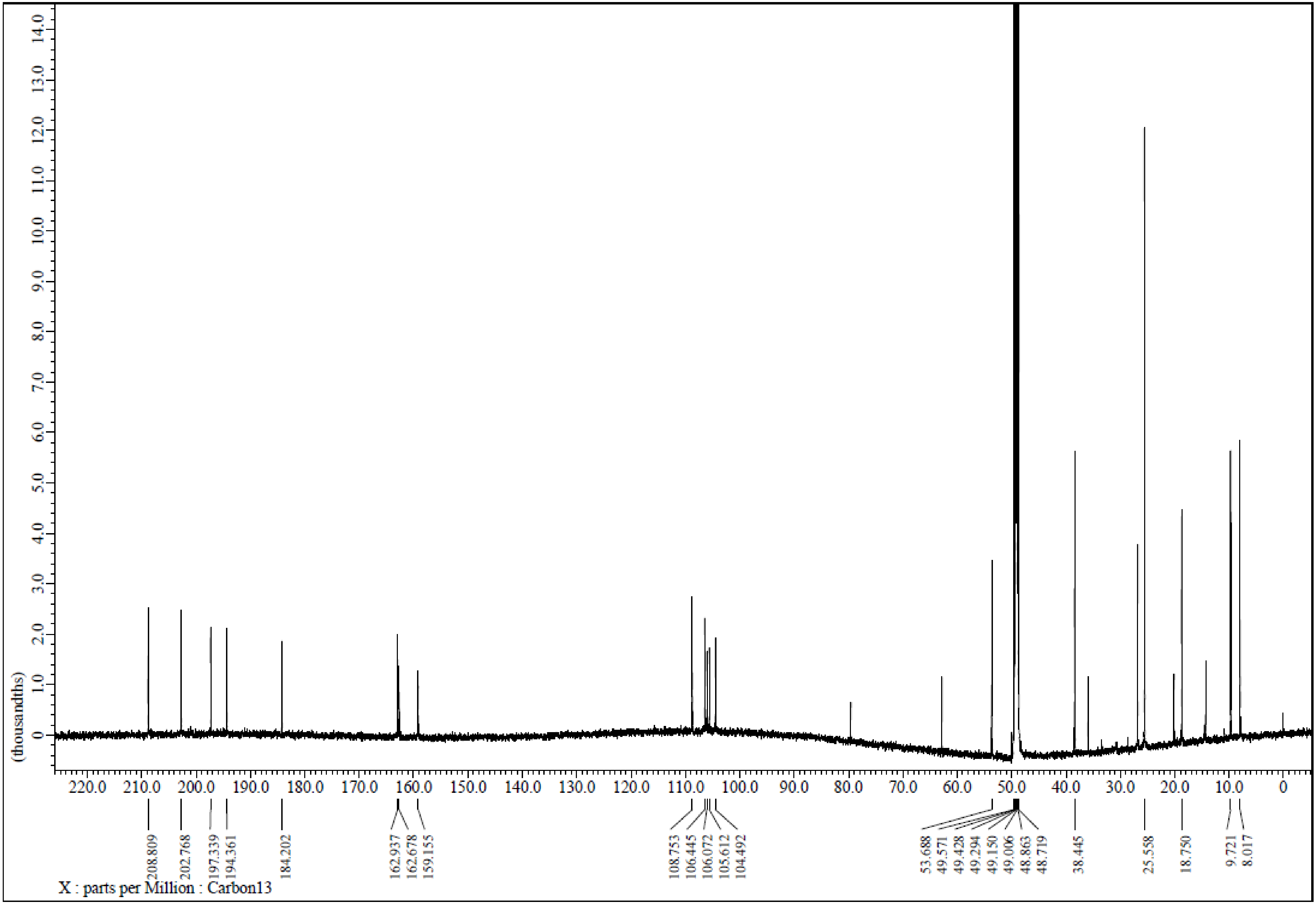

Figure S5. The ^{13}C NMR spectrum of compound **1** (150 MHz, CD_3OD).

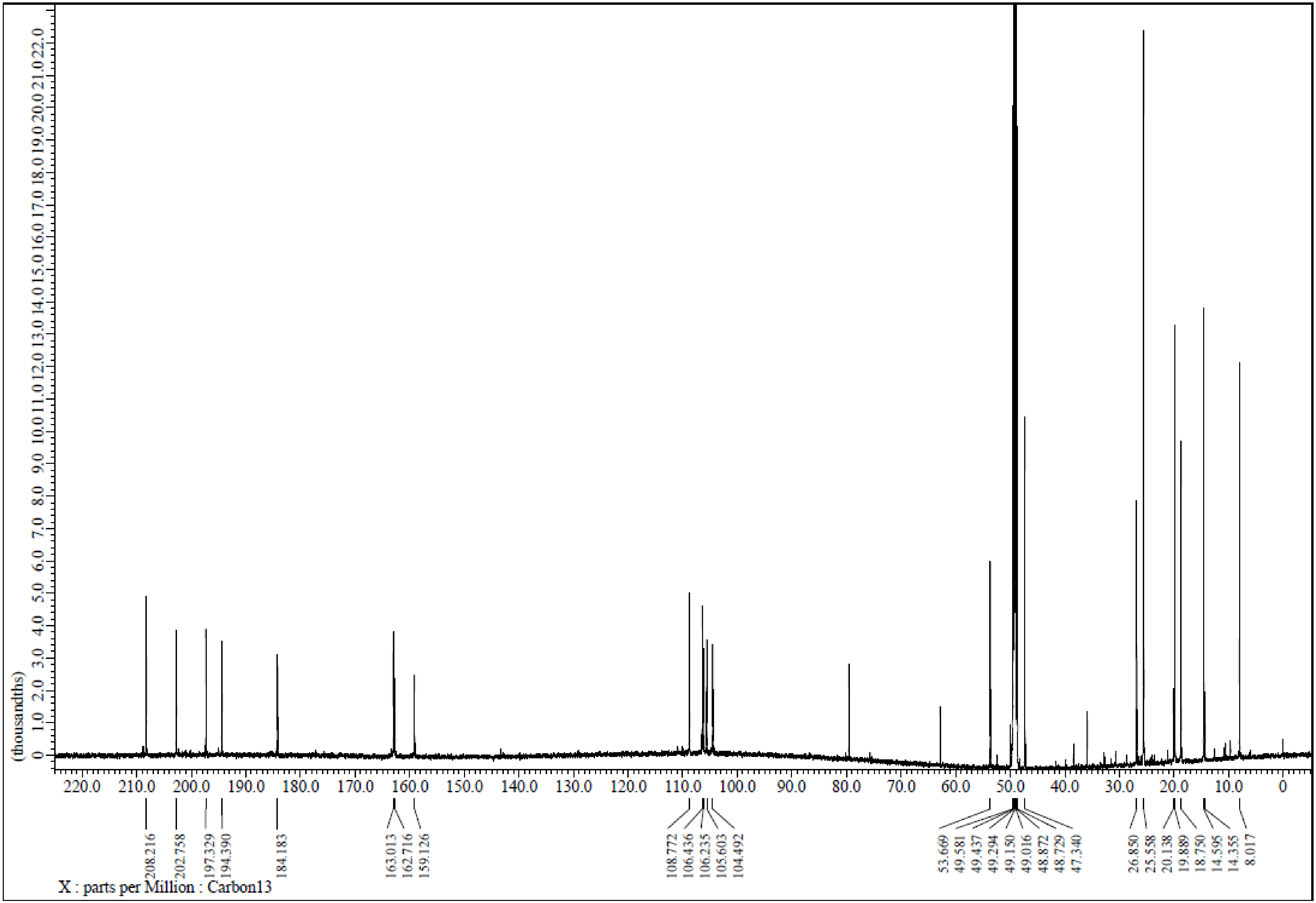

Figure S6. The ^{13}C NMR spectrum of compound **2** (150 MHz, CD_3OD).

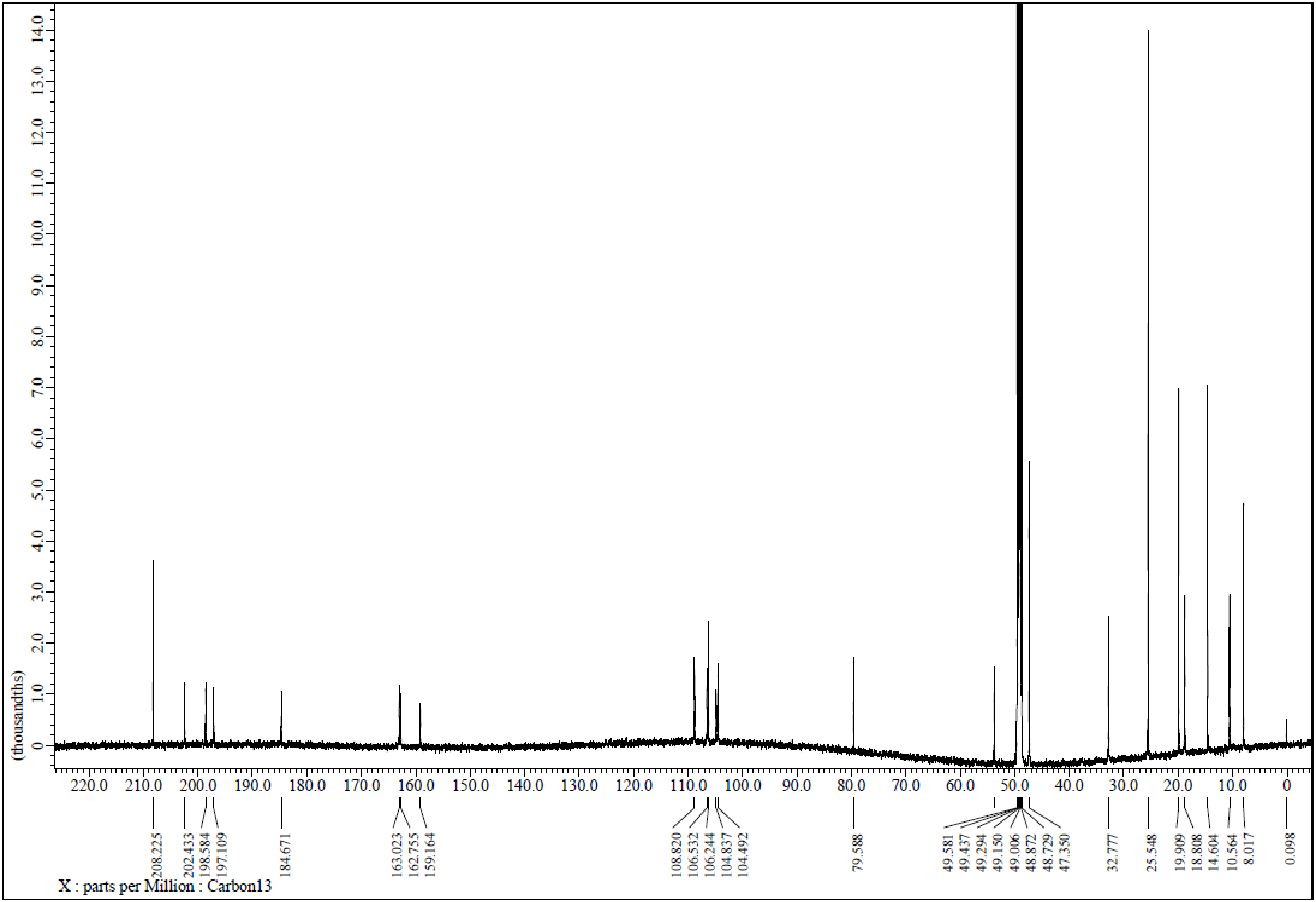

Figure S7. The ^{13}C NMR spectrum of compound **3** (150 MHz, CD_3OD).

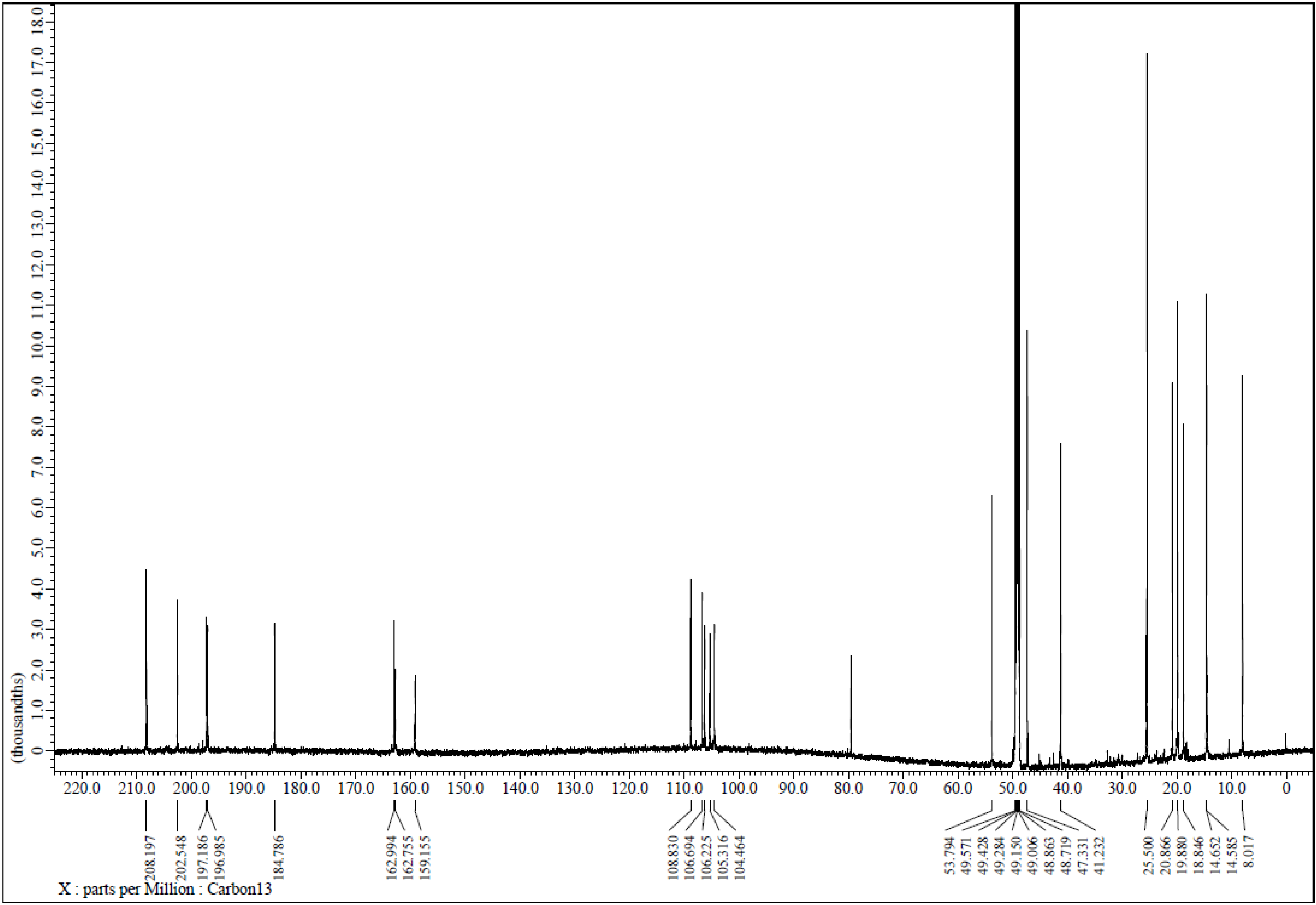

Figure S8. The ^{13}C NMR spectrum of compound **4** (150 MHz, CD_3OD).

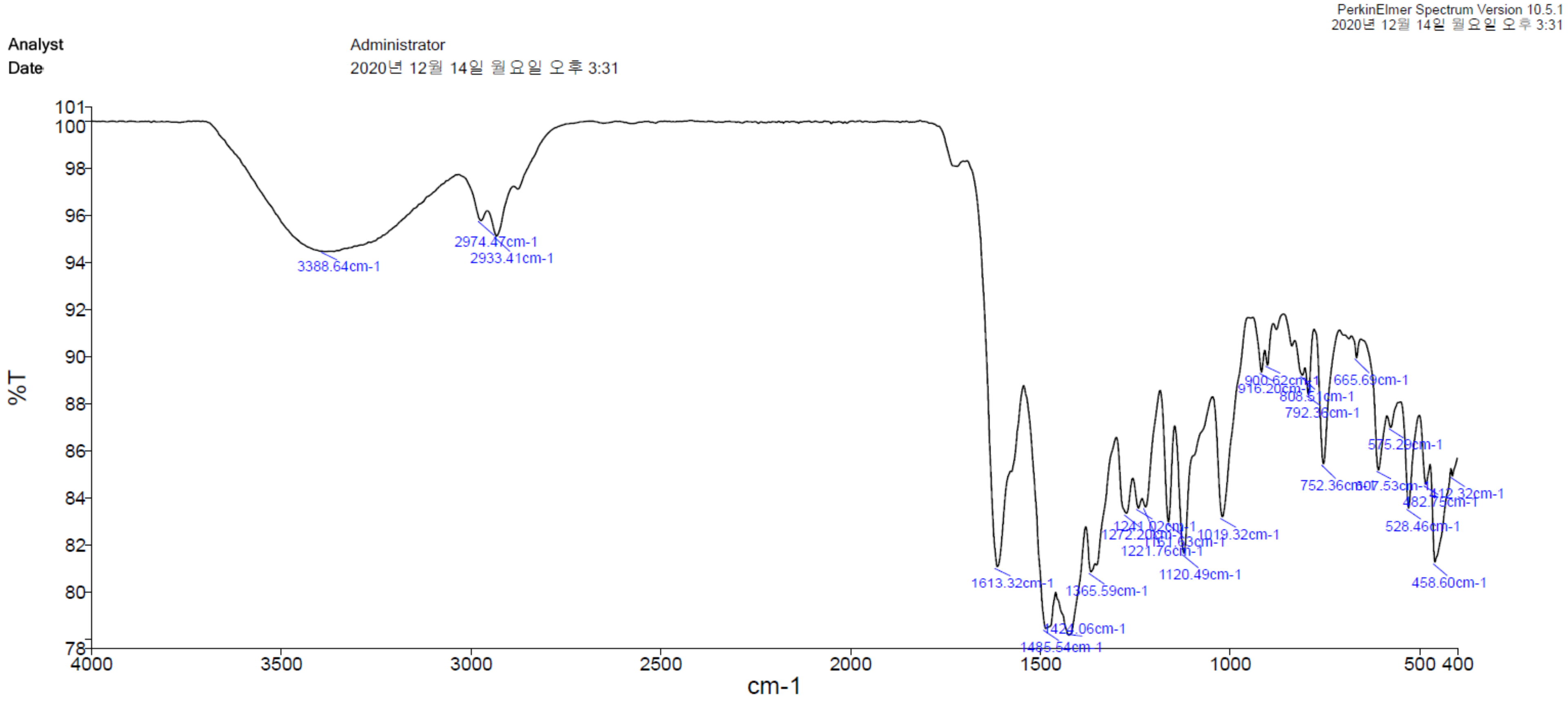

Figure S9. The IR spectrum of compound **1**.

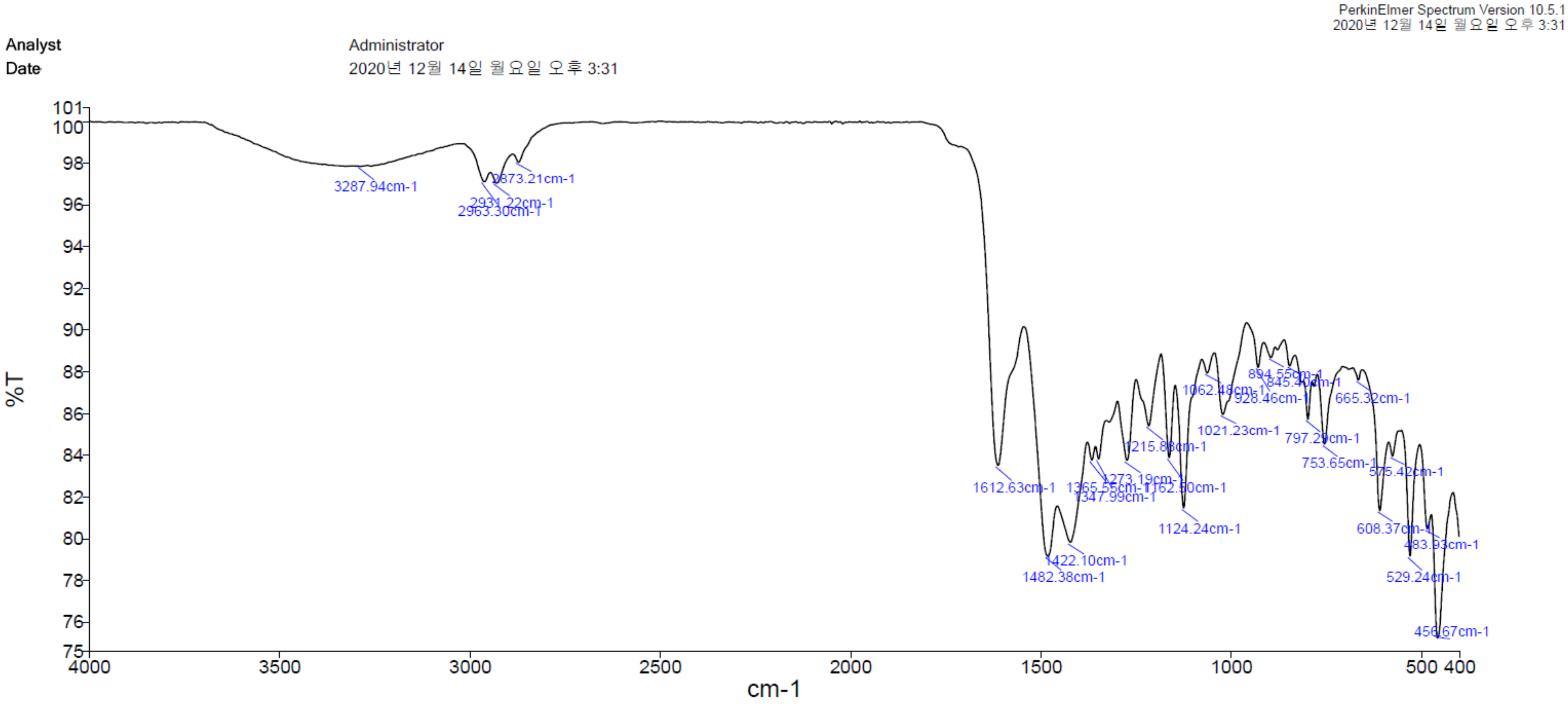

Figure S10. The IR spectrum of compound **2**.

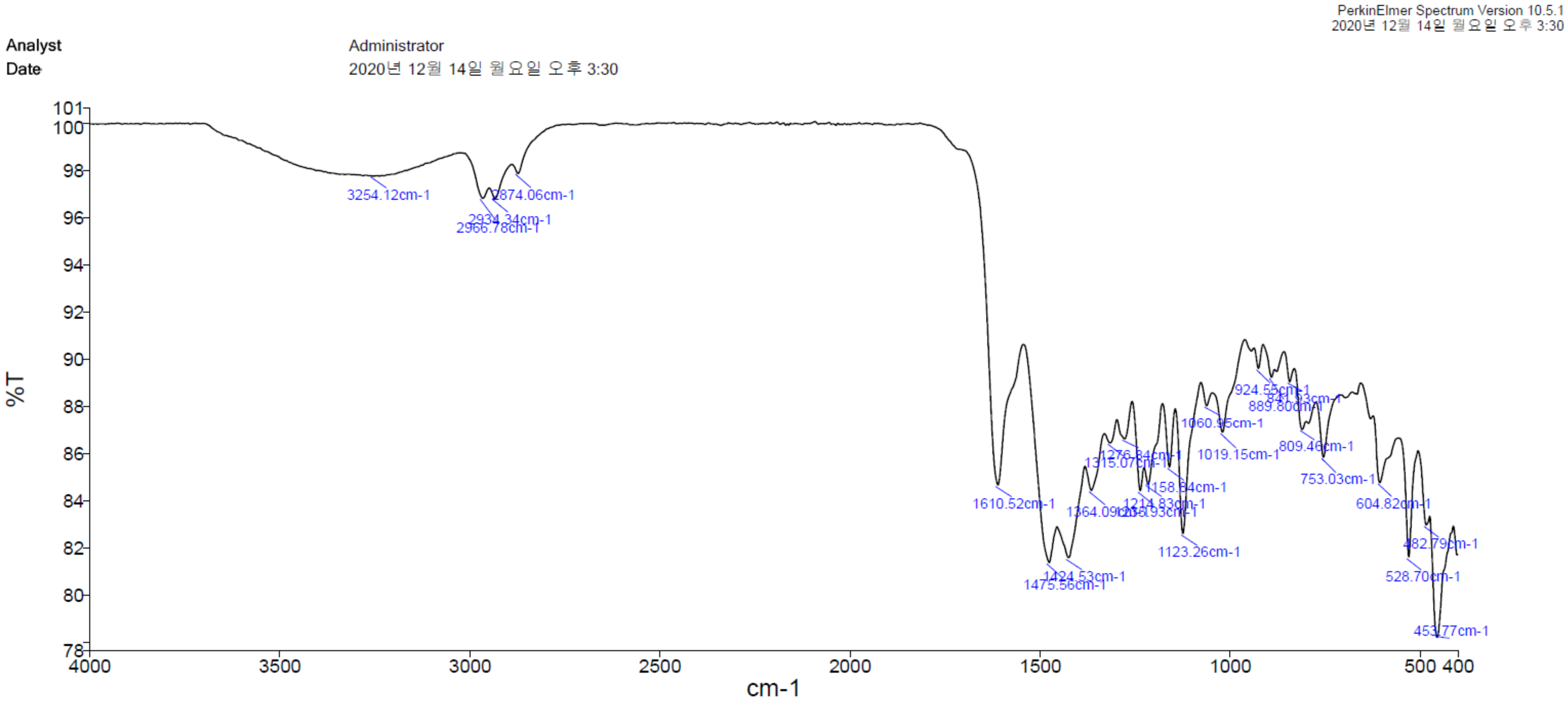

Figure S11. The IR spectrum of compound **3**.

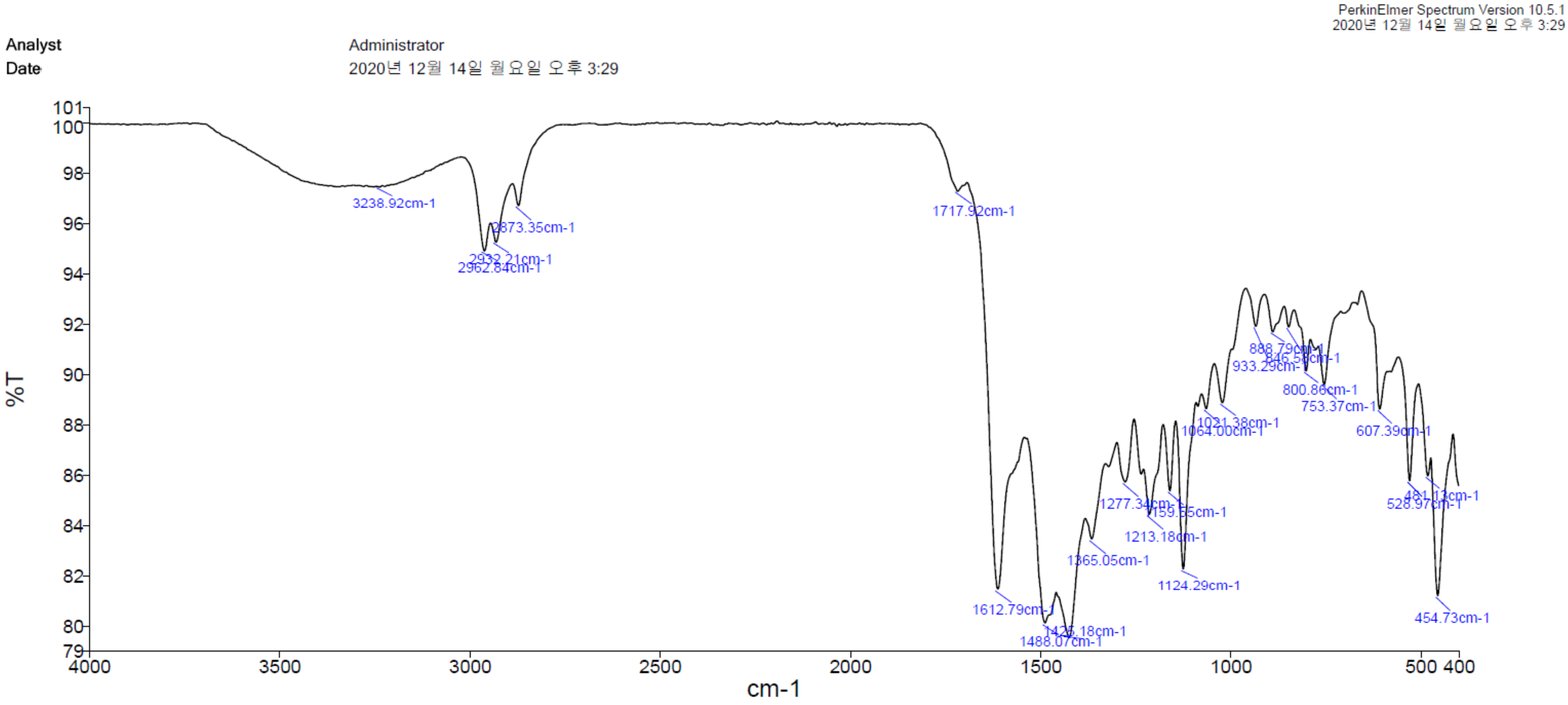

Figure S12. The IR spectrum of compound **4**.

[UV spectrum]

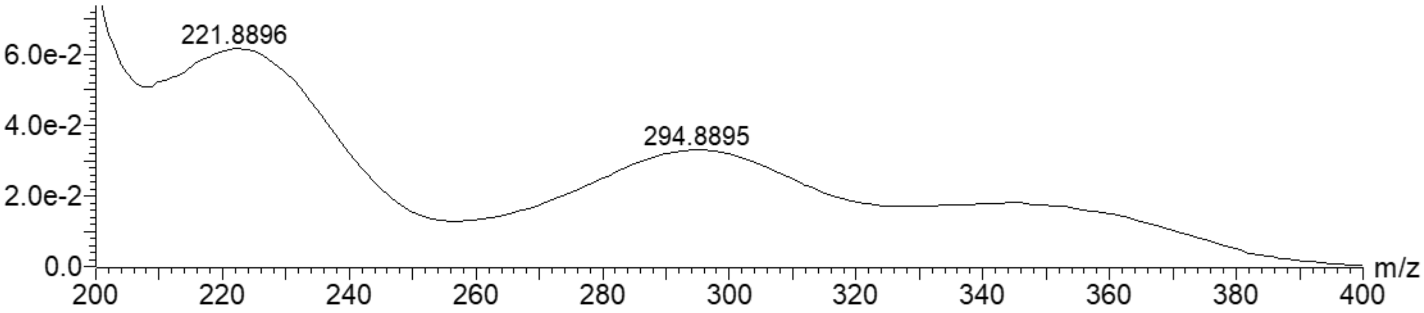

[MS Chromatogram]
negative

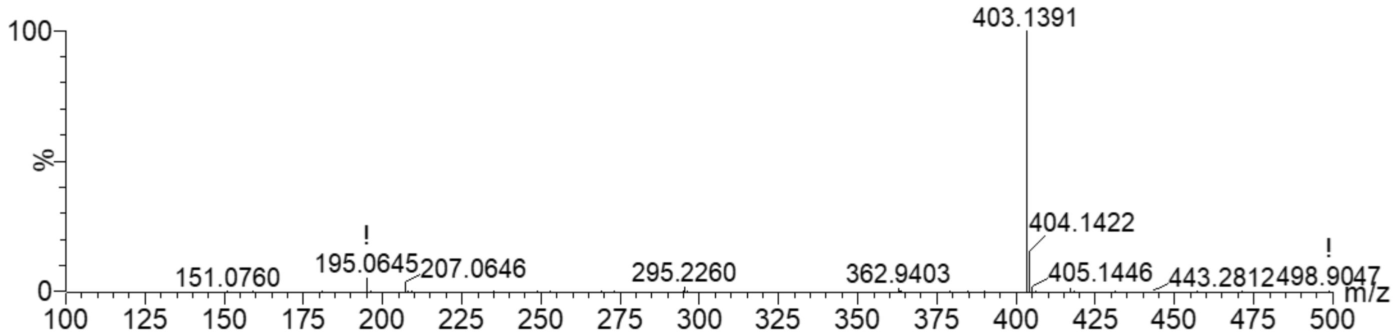

[MS Chromatogram]
positive

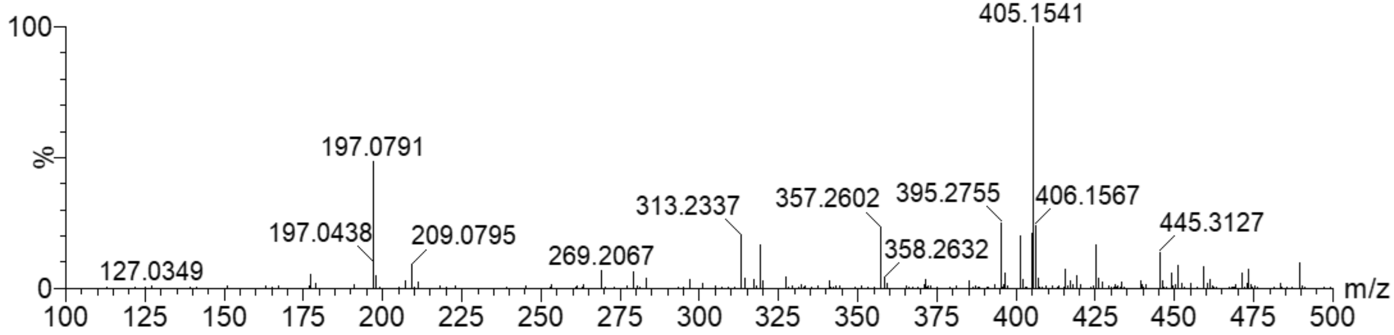

Elemental Composition:
[HR-ESI/MS] negative

Single Mass Analysis
Tolerance = 5.0 PPM / DBE: min = -1.5, max = 50.0
Element prediction: Off
Number of isotope peaks used for i-FIT = 3
Monoisotopic Mass, Even Electron Ions
92 formula(e) evaluated with 1 results within limits (up to 50 closest results for each mass)
Elements Used:

Mass	Calc. Mass	mDa	PPM	DBE	Formula	i-FIT	i-FIT Norm	Fit Conf %	C	H	O
403.1391	403.1393	-0.2	-0.5	10.5	C21 H23 O8	607.0	n/a	n/a	21	23	8

Figure S13. Identification of compound **1** by UPLC-QTof MS.

[UV spectrum]

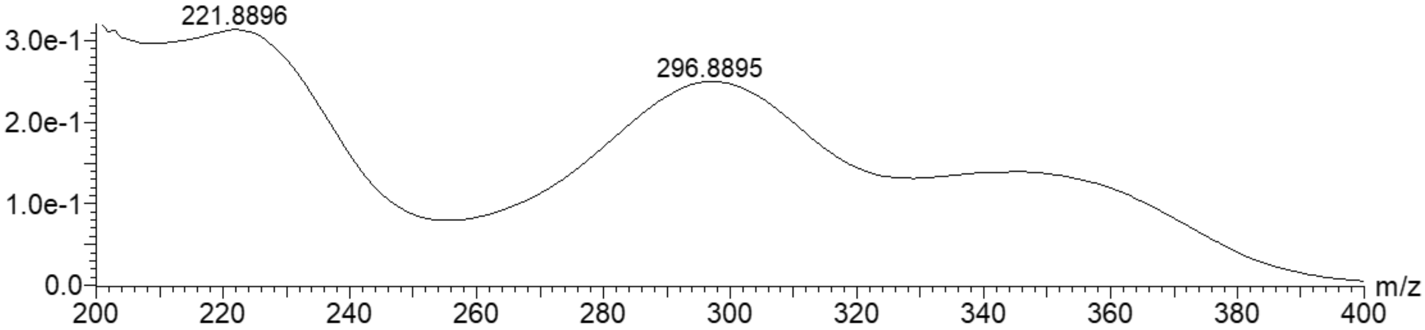

[MS Chromatogram] negative

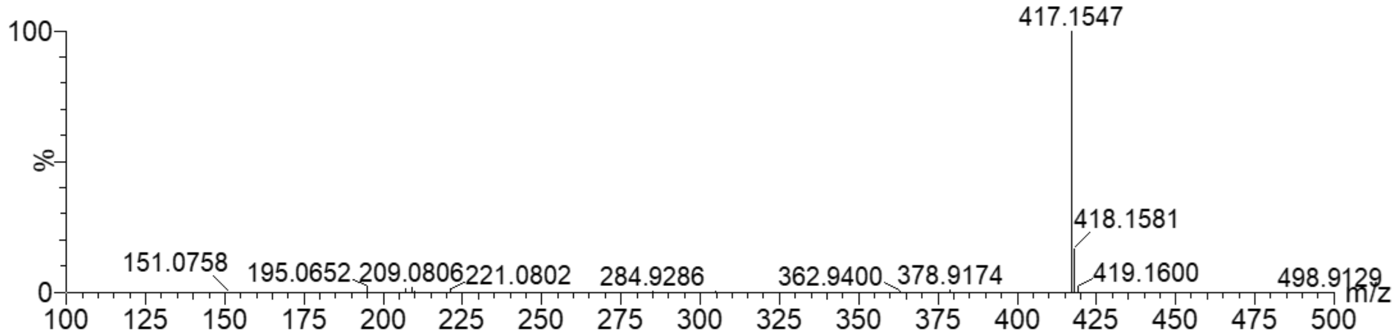

[MS Chromatogram] positive

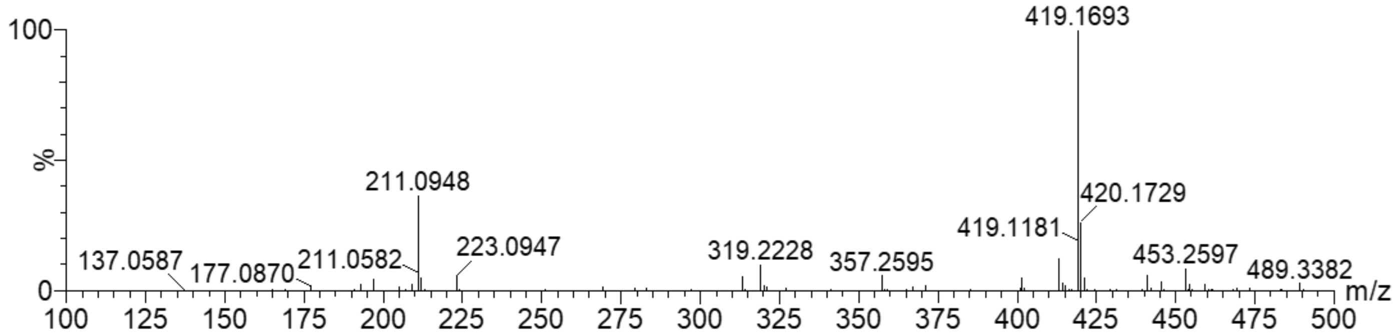

Elemental Composition: [HR-ESI/MS] negative

Single Mass Analysis

Tolerance = 5.0 PPM / DBE: min = -1.5, max = 50.0

Element prediction: Off

Number of isotope peaks used for i-FIT = 3

Monoisotopic Mass, Even Electron Ions

93 formula(e) evaluated with 1 results within limits (up to 50 closest results for each mass)

Elements Used:

Mass	Calc. Mass	mDa	PPM	DBE	Formula	i-FIT	i-FIT Norm	Fit Conf %	C	H	O
417.1547	417.1549	-0.2	-0.5	10.5	C22 H25 O8	693.2	n/a	n/a	22	25	8

Figure S14. Identification of compound **2** by UPLC-QTof MS.

[UV spectrum]

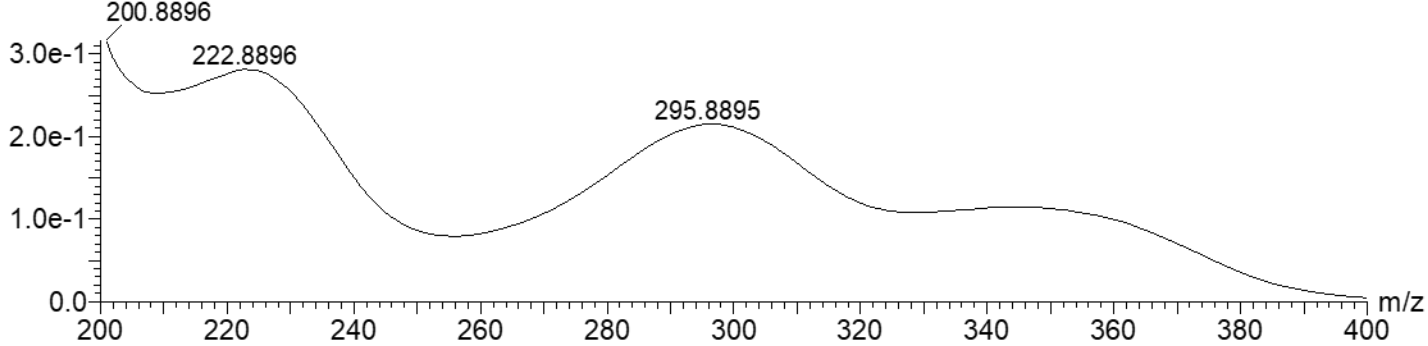

[MS Chromatogram]
negative

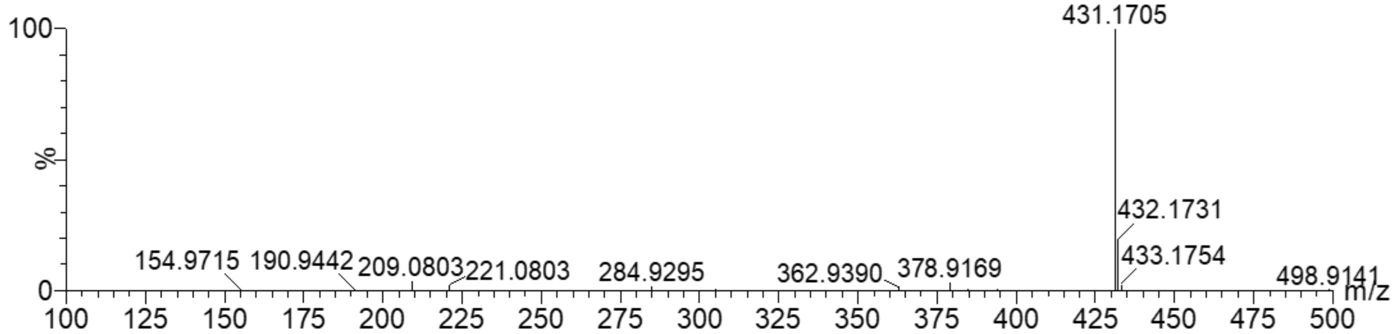

[MS Chromatogram]
positive

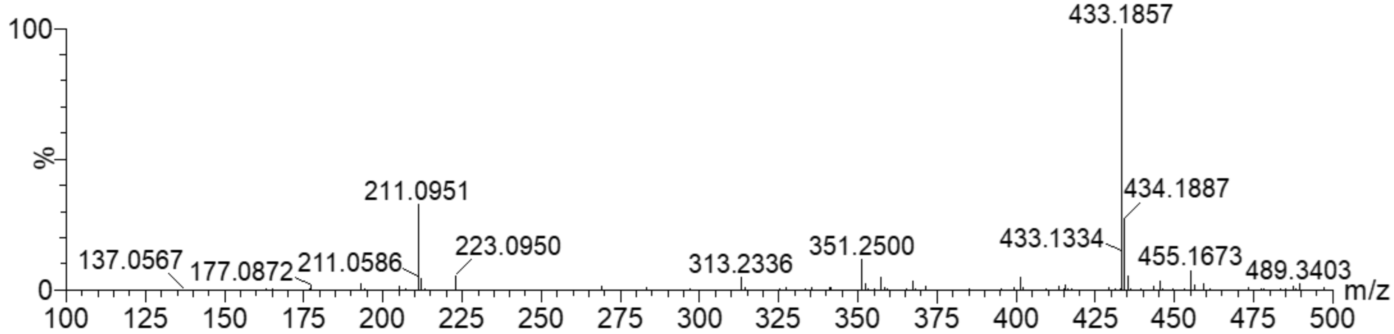

Elemental Composition:
[HR-ESI/MS] negative

Single Mass Analysis

Tolerance = 5.0 PPM / DBE: min = -1.5, max = 50.0

Element prediction: Off

Number of isotope peaks used for i-FIT = 3

Monoisotopic Mass, Even Electron Ions

102 formula(e) evaluated with 1 results within limits (up to 50 closest results for each mass)

Elements Used:

Mass	Calc. Mass	mDa	PPM	DBE	Formula	i-FIT	i-FIT Norm	Fit Conf %	C	H	O
431.1705	431.1706	-0.1	-0.2	10.5	C23 H27 O8	770.4	n/a	n/a	23	27	8

Figure S15. Identification of compound **3** by UPLC-QTof MS.

[UV spectrum]

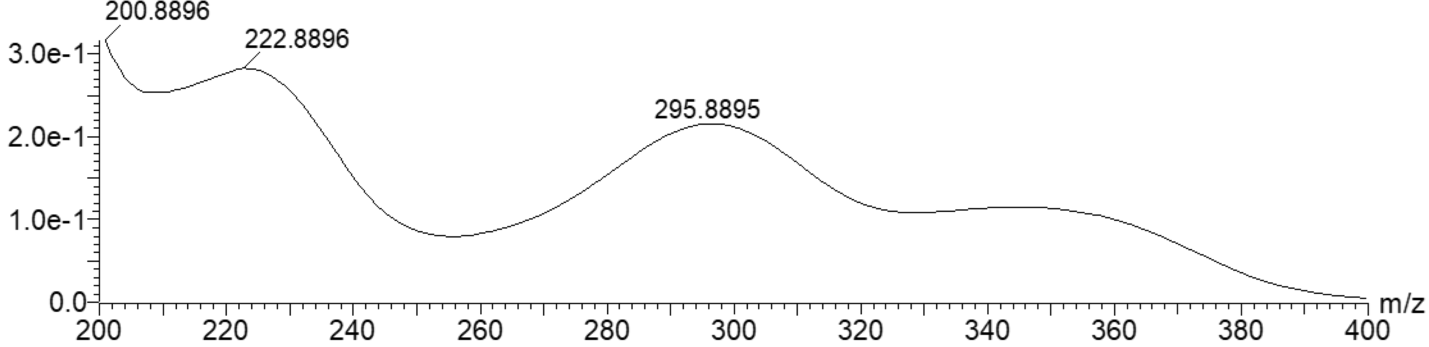

[MS Chromatogram] negative

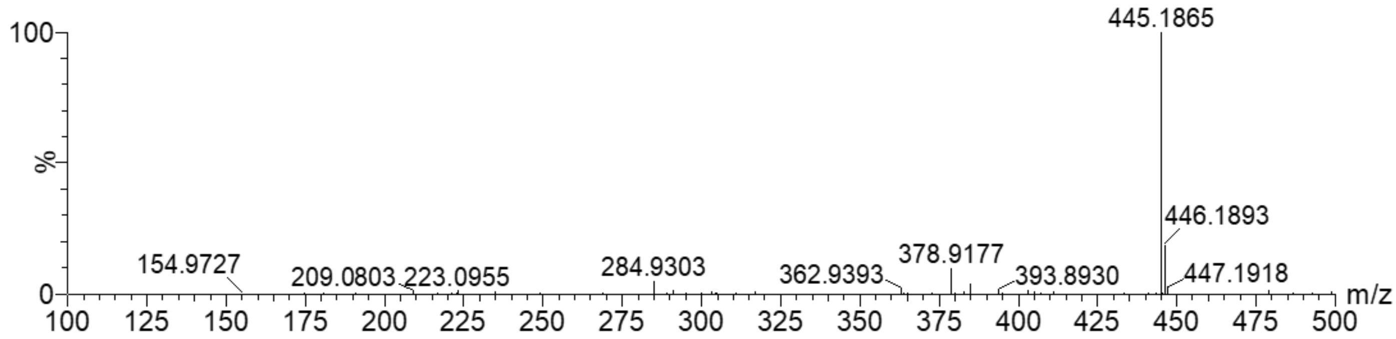

[MS Chromatogram] positive

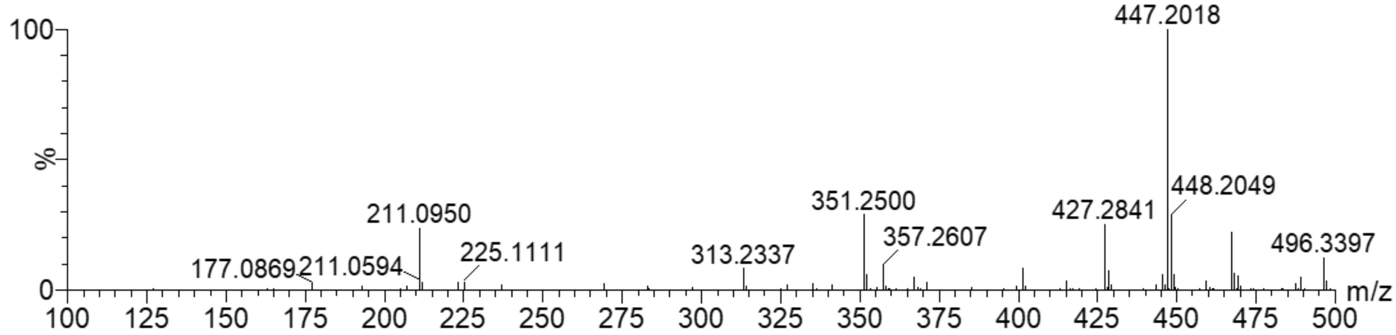

Elemental Composition: [HR-ESI/MS] negative

Single Mass Analysis

Tolerance = 5.0 PPM / DBE: min = -1.5, max = 50.0

Element prediction: Off

Number of isotope peaks used for i-FIT = 3

Monoisotopic Mass, Even Electron Ions

103 formula(e) evaluated with 1 results within limits (up to 50 closest results for each mass)

Elements Used:

Mass	Calc. Mass	mDa	PPM	DBE	Formula	i-FIT	i-FIT Norm	Fit Conf %	C	H	O
445.1865	445.1862	0.3	0.7	10.5	C24 H29 O8	799.7	n/a	n/a	24	29	8

Figure S16. Identification of compound **4** by UPLC-QTof MS.

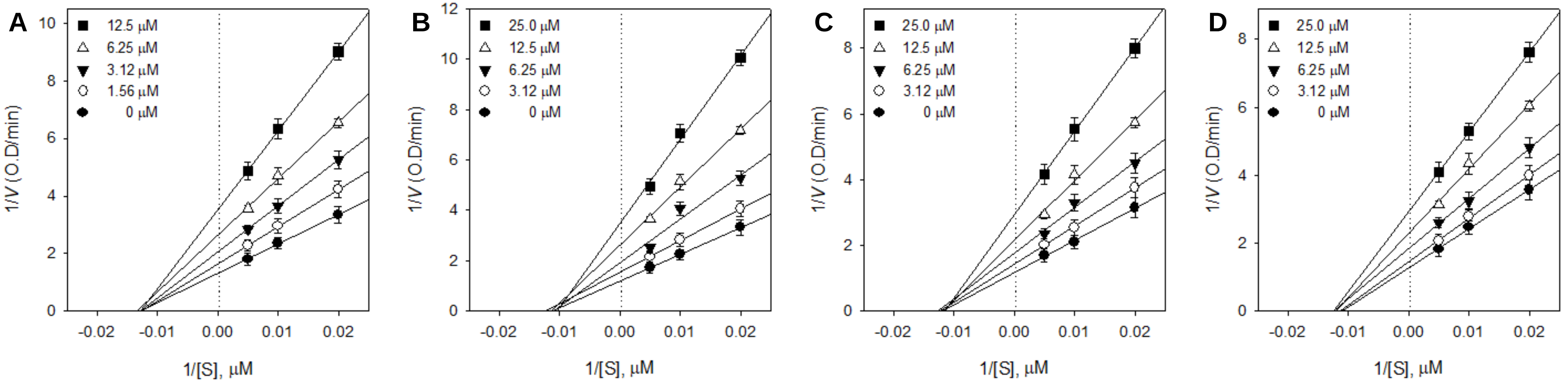

Figure S17. (A−D) Lineweaver-Burk plots of XO inhibition by compounds (**1**−**4**).

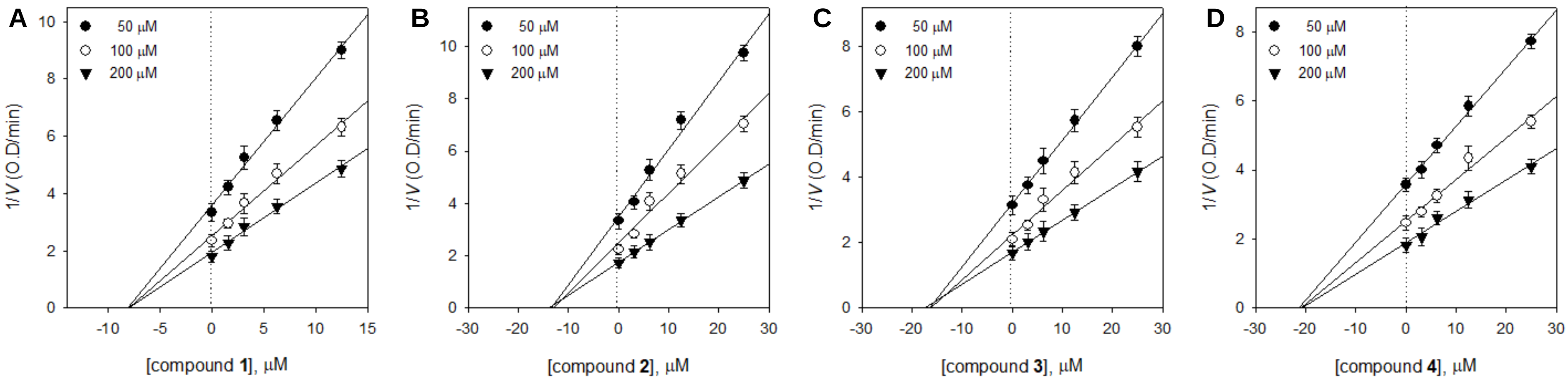

Figure S18. (A−D) Dixon plots of XO inhibition by compounds (**1**−**4**).
